# pH-responsive substrate switching in mycobacterial type VII ESX secretion

**DOI:** 10.1128/msphere.00056-26

**Published:** 2026-04-29

**Authors:** Owen A. Collars, Richard L. Hernandez, Simon D. Weaver, Rebecca J. Prest, Caleb Manu, Gopinath Viswanathan, Rachel M. Cronin, Bradley S. Jones, David M. Tobin, Matthew M. Champion, Patricia A. Champion

**Affiliations:** 1 Department of Biological Sciences, University of Notre Dame 167056, Notre Dame, Indiana, USA; 2 Department of Chemistry and Biochemistry, University of Notre Dame 6111, Notre Dame, Indiana, USA; 3 Department of Molecular Genetics & Microbiology, Duke University School of Medicine 12277 , Durham, North Carolina, USA; 4 Department of Integrative Immunobiology, Duke University School of Medicine 12277 , Durham, North Carolina, USA; 5 Department of Cell Biology, Duke University School of Medicine 12277 , Durham, North Carolina, USA; University of Minnesota Twin Cities, Minneapolis, Minnesota, USA

**Keywords:** *Mycobacterium marinum*, ESX-1, Type VII, pH, secretion

## Abstract

During infection, pathogenic mycobacteria reside within phagosomes of varying acidity based on the macrophage activation state. The ESX-1 secretion system (early secreted antigen 6 kilodaltons [ESAT-6] system 1) delivers protein virulence factors essential for phagosome lysis, facilitating infection. The mechanisms underlying ESX-1 lytic activity in heterogeneous environments remain unknown. Here, we show that the canonical Type VII secretion system, ESX-1, orchestrates substrate switching in response to different environments. Growing *Mycobacterium marinum* at acidic pH resulted in substrate switching *in vitro*. Substrate switching was accompanied by significant changes to the levels of ESX-1 substrate transcripts and to the levels of both ESX-1 substrates and chaperones at the protein level. We showed that specific ESX-1 transcripts were significantly upregulated *in vivo* and that distinct substrate sets are required in an acidic infection model.

**IMPORTANCE:**

Pathogenic mycobacteria cause chronic and acute disease. Mycobacterial pathogens promote infection by transporting bacterial proteins into the host using ESX/Type VII secretion systems. The ESX-1 system secretes proteins into the phagosome that release the bacteria into the cytoplasm and promote bacterial survival in the macrophage. We show that *Mycobacterium marinum*, an animal pathogen and model for studying ESX-1 and tuberculosis, switches which ESX-1 proteins are secreted in response to acidic pH, an infection relevant signal. We demonstrate that protein secretion reflects changes in substrate transcripts and in substrate and chaperone protein levels. Finally, we leveraged two infection models to support that ESX-1 substrate switching likely occurs during infection. Our findings support a model in which mycobacterial pathogens use different proteins to lyse macrophage phagosomes of different pH.

## INTRODUCTION

*Mycobacterium tuberculosis*, the cause of human tuberculosis, transiently resides in the macrophage phagosome during infection (1–3). During this time, the bacteria sense the phagosomal pH and mediate a transcriptional response (4). The pH of the phagosome is dependent on the activation state of the macrophage (5). In naïve macrophages, mycobacteria arrest phagosome maturation, restricting the pH to a slightly acidic 6.0–6.5 (6). In activated macrophages, the phagosome fuses with the lysosome, relegating the mycobacteria to an acidic compartment with a pH of 4.5–5.0 (7).

ESX-1 is the canonical Type VII secretion system and is essential for mycobacterial infection because it promotes phagosome escape (8–11). ESX-1 secretes protein substrates to the mycobacterial cell surface and into the environment (8, 12–17). Some ESX-1 substrates have been detected in the phagosome during macrophage infection (18), and several ESX-secreted proteins are presented as MHC-1 peptides (19). One or more ESX-1 substrates, along with virulence lipids, likely damage the phagosomal membrane, allowing mycobacterial pathogens access to the macrophage cytoplasm (11, 20–22). Cytoplasmic access activates host response pathways which result in macrophage lysis and bacterial spread (10, 23–27). It is unknown if the ESX-1 system differentially responds to changing environments during infection.

*Mycobacterium marinum,* a pathogen that causes a tuberculosis-like disease in poikilothermic animals, is an established model for studying *M. tuberculosis* infection biology (28–31). The ESX-1 system is functionally conserved between *M. marinum* and *M. tuberculosis* (32, 33). Importantly, *M. marinum* has been instrumental in dissecting the molecular function of the ESX-1 secretion system.

**Fig 1:**
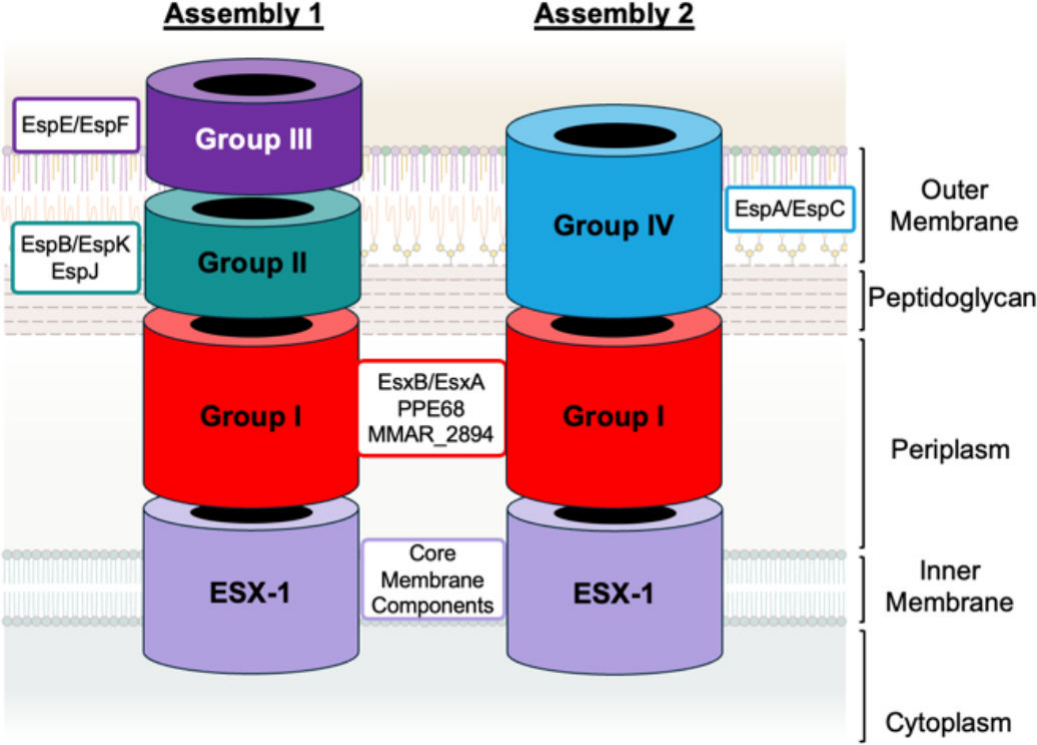
Model of proposed ESX-1 secretion assemblies. Model based on proteo-genetic data from (34). Substrates and components drawn as rings for simplicity based on reports of ring structures for EspB (36) and the ESX-1 membrane complex (37–40). Core membrane components include EccA-E (8, 41).

The precise machinery required for ESX-1 substrate translocation across the mycobacterial cell envelope is unknown. The ESX-1 substrates require each other for secretion (8, 16). Using proteomics and genetics, we proposed an “inside out” model for ESX-1 protein secretion in which secreted substrates span the mycobacterial cell envelope, promoting the secretion of later substrates to the surface and out of the mycobacterial cell (Fig. 1). We identified four substrate groups (Groups I, II, III, and IV [34]). The Group I substrates were required for the secretion of the other substrate groups. The Group I and Group II substrates were required for Group III substrate secretion. The Group II and Group III substrates were not required for Group IV substrate secretion. However, the secretion of Group IV substrates was higher from strains lacking the Group II or Group III substrates (34). We hypothesized that the Group II/III substrates and the Group IV substrates are secreted in opposition, potentially forming distinct substrate “assemblies” that function under different environmental conditions (Fig. 1). The different assemblies would require *M. marinum* to switch between secreting the Group II/III and the Group IV substrates. Although substrate switching is well defined in Type III secretion systems in Gram-negative bacteria, it has not been demonstrated for ESX secretion systems. For Type III systems, the destination of the secreted substrates acts as the signal for substrate switching. For example, in *Salmonella*, the neutral pH of the macrophage cytosol is the signal that promotes the switch from secretion system components to effectors (35). In this study, we leverage *M. marinum* to demonstrate ESX-1 substrate switching in response to physiologically relevant pH changes during standard laboratory growth and during infection.

## RESULTS

### The ESX-1 substrates are required for hemolytic activity change in response to environmental pH

The mycobacterial ESX-1 system secretes protein substrates into the macrophage phagosome (18). A unique aspect of the *M. marinum* model is that ESX-1 lytic activity can be measured *in vitro. M. marinum* lyse red blood cells in a contact-dependent, ESX-1-dependent manner (42, 43). Hemolytic activity simplifies measuring ESX-1 lytic activity because it does not require a host cell and allows synchronization of lysis. We previously reported that the deletion of individual substrate genes from Groups I, II, or III significantly reduced or abrogated *M. marinum* hemolytic activity following growth under standard conditions. Hemolytic activity was restored by genetic complementation (17, 34, 44). The deletion of individual Group IV genes did not impact hemolytic activity (34).

To determine if hemolytic activity correlates with the phagolytic activity of the ESX-1 system, we measured cytoplasmic bacteriolysis of *M. marinum* during macrophage infection. We previously adapted a luciferase reporter from *L. monocytogenes* (45) that measures lysis of *M. marinum* cells in the cytoplasm (bacteriolysis) during macrophage infection (46). Only *M. marinum* strains that can escape the phagosome lyse in the macrophage cytoplasm, allowing the reporter plasmid to be translocated to the nucleus where the luciferase genes are expressed. *M. marinum* strains lacking a functional ESX-1 system are retained in the phagosome, no bacteriolysis occurs in the cytoplasm and the luciferase genes are not expressed. A similar reporter was used to demonstrate ESX-1 dependent cytoplasmic access of *M. tuberculosis* during macrophage infection (47). As shown in Fig. S1A, a measurable population of *M. marinum* lysed in the macrophage cytoplasm resulting in luciferase detection, consistent with our prior report (46). The luciferase levels measured with the WT strain varied between infections, likely resulting from a lack of synchronized phagosome lysis. The Δ*eccCb_1_* strain lacks the ESX-1 membrane complex and is retained in the phagosome (8, 11, 48). Infection with the Δ*eccCb_1_* reporter strain resulted in a significant reduction in luciferase levels relative to infection by the WT reporter strain (consistent with [46]). We tested *M. marinum* strains lacking substrate genes representative from each group. The deletion of substrate genes in Groups I, II, and III resulted in luciferase levels that were not significantly different from infection with the Δ*eccCb_1_* reporter strain (Fig. S1B through F). These data support that the non-hemolytic *M. marinum* strains lacking Group I, II, or III substrates (34) did not undergo cytoplasmic bacteriolysis similar to the phagosome localized Δ*eccCb_1_* strain. Complementation significantly increased luciferase levels relative to the Δ*eccCb_1_* strain in each case. Infection with the Δ*espC* (Group IV) reporter strain resulted in WT levels of luciferase that increased with complementation (Fig. S1G). These data support that the hemolytic *M. marinum* strains lacking Group IV substrates (34) exhibited bacteriolysis similar to the WT strain which accesses the cytoplasm. Together, our data support that ESX-1-dependent hemolytic activity correlates well with ESX-1-dependent phagolytic activity during macrophage infection by *M. marinum*.

**Fig 2:**
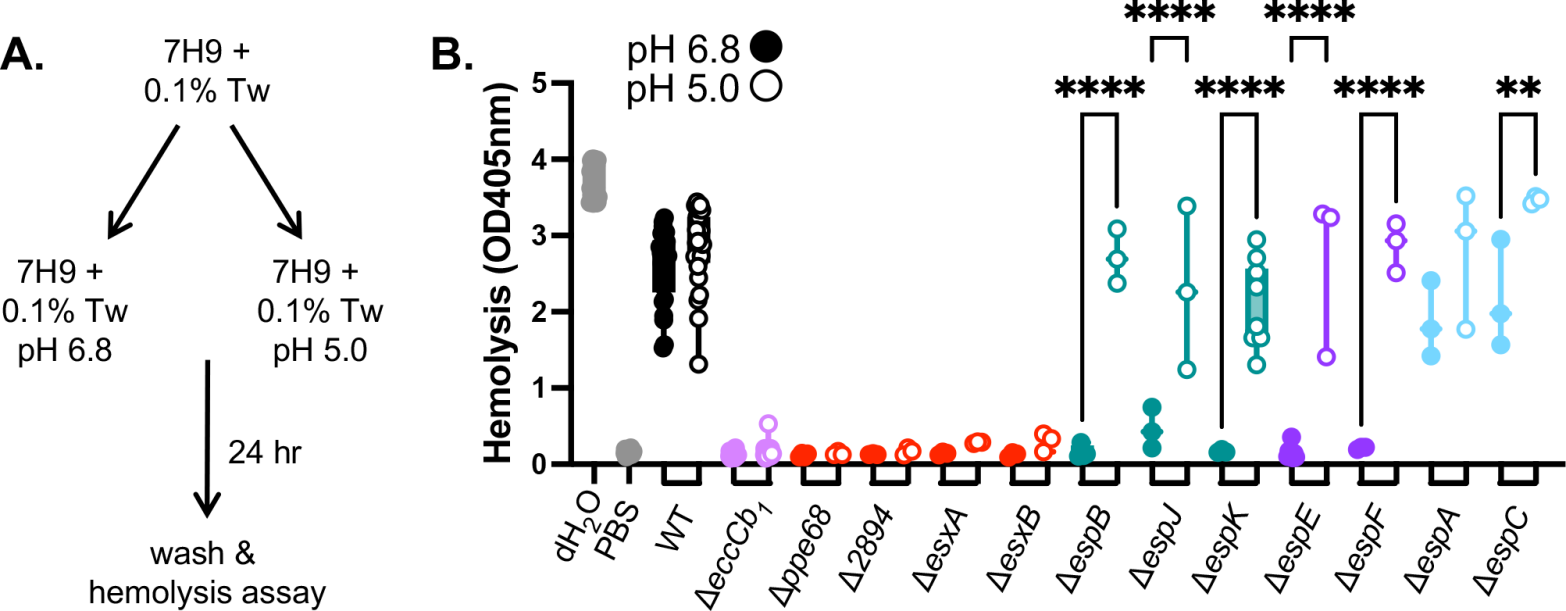
Exposure to acidic pH changes the requirements for hemolytic activity. (**A**) Workflow for acid stress exposure prior to hemolysis assay. Tw: Tween-80. (**B**) Hemolytic activity of *M. marinum* strains bearing single deletions at slightly acidic pH 6.8 (solid circles) or acidic pH 5 (open circles). Data points represent one biological replicate, which is the mean of three technical replicates. Significance was determined using a one-way ordinary ANOVA (*P*< 0.0001) followed by a Tukey’s multiple comparison test. ****P < 0.0001, **P = 0.0023. The double deletion strains were confirmed using PCR (Fig. S2), followed by targeted DNA sequencing.

To determine if ESX-1 switches substrates, we tested if the requirements for specific substrates in ESX-1 mediated hemolytic activity changed in response to exposure to media buffered to different pHs. We reasoned that the requirements for hemolysis might change if substrate secretion switches in response to acidic pH. Using *M. marinum* strains with unmarked deletions of the known ESX-1 substrate genes (34), we exposed cultures to acidic (pH 5.0) or slightly acidic (pH 6.8) MOPS buffered 7H9 media for 24 h (Fig. 2A). We washed the bacteria and measured the hemolytic activity of the *M. marinum* strains. As shown in Fig. 2B, the WT *M. marinum* strain lysed RBCs following growth at pH 6.8 (filled circles) or pH 5.0 (open circles). Water and phosphate-buffered saline (PBS) were cell-free positive and negative controls for hemolysis, respectively. *M. marinum* strains with deletions in the membrane complex genes (*eccCb_1_,* light purple) or in the Group I substrate genes (*esxA, esxB, ppe68,* or *MMAR_2894,* red) were non-hemolytic under both conditions tested. These data indicate that the ESX-1 membrane complex and the Group I substrates were essential for hemolytic activity following either growth condition. *M. marinum* strains with deletions of the genes encoding Group II (teal), or Group III (purple) ESX-1 substrates exhibited significantly increased hemolytic activity following growth at pH 5.0, compared to growth at pH 6.8 (Fig. 2B). These data support that Group II and Group III substrates were required for hemolysis under slightly acidic conditions, but dispensable at acidic conditions. *M. marinum* strains lacking the genes encoding the Group IV substrates (light blue) exhibited hemolytic activity following growth under either condition (Fig. 2B). The Δ*espC* strain was significantly more hemolytic following growth at pH 5.0 as compared to pH 6.8. These data support that the requirements for *M. marinum* hemolytic activity change in response to the pH of the growth media.

### Group III and Group IV substrates can promote hemolysis in *M. marinum*

**Fig 3:**
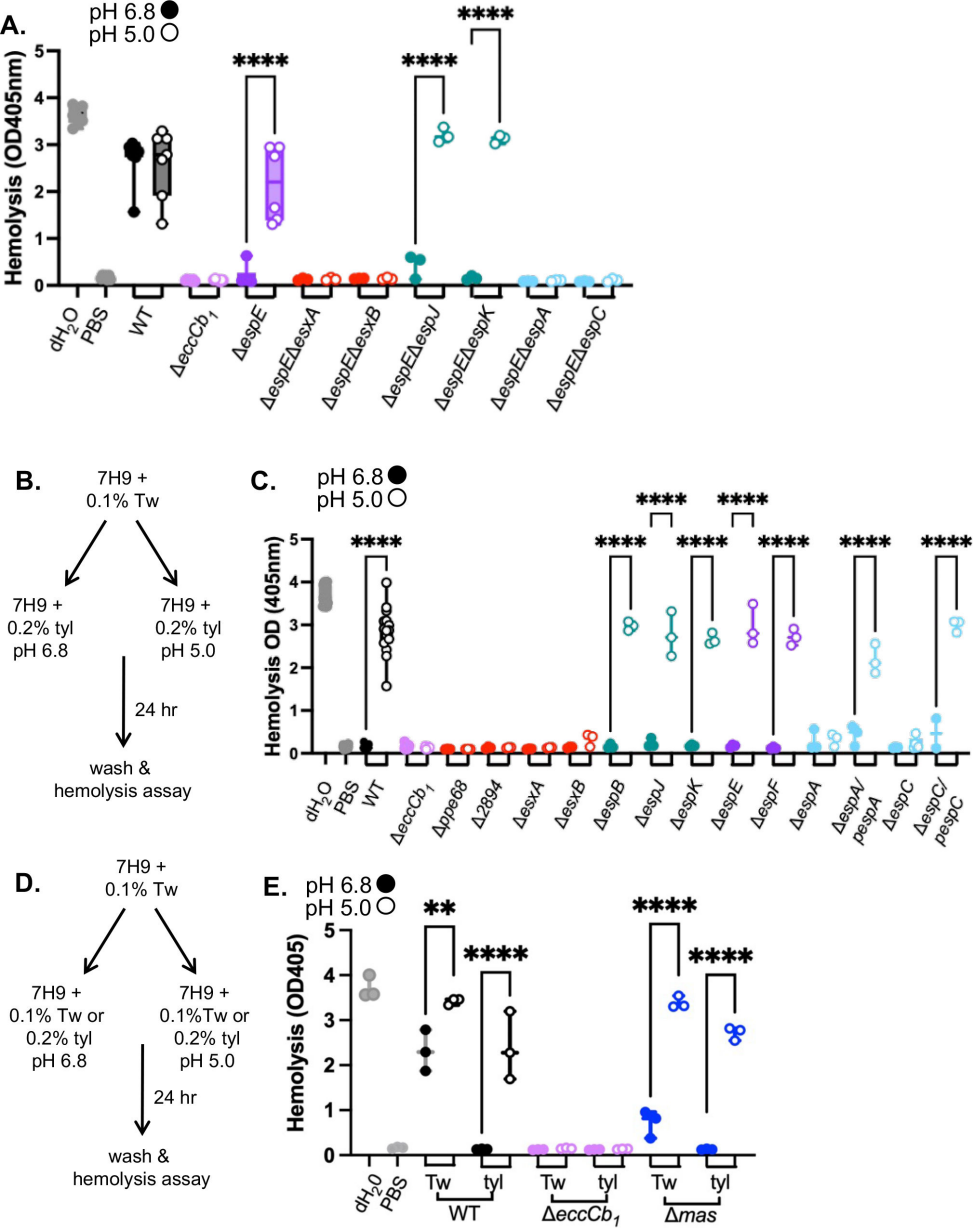
Group IV substrates are required for hemolysis in the absence of Group III substrates at pH 5. (**A**) Hemolytic activity of *M. marinum* strains lacking the *espE* gene (Group III) and representative substrate genes from each class following growth at pH 6.8 (closed circles) or pH 5.0 (open circles). Data points are individual biological replicates, which are the means of technical triplicate readings. Significance was determined using a one-way ordinary ANOVA (*P* < 0.0001) followed by a Tukey’s multiple comparison test. ****P < 0.0001. (**B**) Schematic of growth conditions for panel B., tyl = tyloxapol. (**C**) Hemolytic activity of *M. marinum* strains following exposure to tyloxapol and pH changes. Data points are individual biological replicates, which are the means of technical triplicate readings. Significance was determined using a one-way ordinary ANOVA (*P* < 0.0001) followed by a Tukey’s multiple comparison test. ****P < 0.0001. (**D**) Schematic of growth conditions for panel B. tyl = tyloxapol. Tw = Tween-80. (**E**) Hemolytic activity of *M. marinum* strains following exposure to tyloxapol or Tween-80 and pH changes. Data points are individual biological replicates, which are the means of technical triplicate readings. Significance was determined using a one-way ordinary ANOVA (*P*< 0.0001) followed by a Tukey’s multiple comparison test. **P = 0.0027, ****P < 0.0001.

The data in Fig. 2 suggested that in the absence of the Group II/ III substrates, another group of ESX-1 substrates promoted hemolysis at pH 5.0 (Fig. 1). We generated a collection of *M. marinum* strains lacking the *espE* Group III substrate gene in combination with deletions of the other ESX-1 substrate genes (Table S1; Fig. S2). EspE directly interacts with EspF (49). The deletion of the *espE* gene prevents the secretion of the other Group III protein, EspF, from *M. marinum* (34, 44). We tested the resulting double deletion strains for hemolytic activity following growth at pH 6.8 and pH 5.0. In agreement with Fig. 2B, the Δ*espE* strain was significantly more hemolytic following growth at pH 5.0 than at pH 6.8 (Fig. 3A). The hemolytic activity of the Δ*espE*Δ*esxA* and Δ*espE*Δ*esxB* strains (Group I, red) or Δ*espE*Δ*espA* and Δ*espE*Δ*espC* (Group IV, blue) was not significantly different following growth at pH 6.8 or pH 5.0. These data suggest that the Group I substrates, EsxA and EsxB, and the Group IV substrates, EspA and EspC, are required for the hemolytic activity of the Δ*espE* strain following growth at pH 5.0. Conversely, the Δ*espE*Δ*espJ* or Δ*espE*Δ*espK* strains (Group II, teal) were non-hemolytic following growth at pH 6.8, but were significantly more hemolytic following growth at pH 5.0, similar to the Δ*espE* strain. These data suggest that the Group II substrates, EspJ and EspK, are dispensable for the hemolytic activity of the Δ*espE* strain when grown at pH 5.0. Together, these data suggest that the substrates required for the hemolytic activity of *M. marinum* switch in response to growth at acidic pH. Moreover, these data support that there are at least two sets or assemblies of ESX-1 substrates, Group II/III and Group IV, substrates that can promote the lytic activity of *M. marinum* (Fig. 1).

In Type III secretion, substrate competition for the secretory machinery is an important aspect of substrate switching (50–54). The deletion of individual or multiple substrate genes could impact the mechanism of secretion or substrate selection. We sought to conditionally inhibit hemolytic activity without the genetic deletion of substrate genes to define which substrates were essential for hemolysis following growth under acidic conditions.

We previously demonstrated that growth of *M. marinum* in tyloxapol blocked hemolytic activity by significantly reducing the secretion of specific Group I, II, and III substrates but increased the secretion of the Group IV ESX-1 substrates (55). Based on our prior work, we reasoned that tyloxapol may be a chemical tool to block hemolysis mediated by Group II/Group III substrates, allowing us to test if the Group IV substrates mediate hemolysis in a wild-type genetic background (55). We grew the WT and Δ*eccCb_1_ M. marinum* strains, and those lacking individual substrate genes, in 7H9 with 0.1% Tween-80 (Fig. 3B). We collected and washed the resulting *M. marinum* cells and resuspended them in 7H9 media buffered to either pH 6.8 or pH 5.0, with 0.2% tyloxapol for 24 h. We washed the cells and measured hemolytic activity. As shown in Fig. 3C, the WT strain, although non-hemolytic following growth at pH 6.8 [closed circles, consistent with reference 55], exhibited significantly increased hemolysis following growth at pH 5 (open circles) in the presence of tyloxapol similar to the cell-free water control. *M. marinum* strains lacking the *eccCb_1_* gene, the Group I substrate genes (*esxA, esxB, ppe68* or *MMAR_2894,* red), or the Group IV substrate genes (*espA or espC*, light blue) were non-hemolytic under either condition tested. Constitutive expression of the Group I genes (Fig. S3) or the Group IV genes in their respective deletion strains significantly restored hemolysis following growth in tyloxapol at pH 5, but not at pH 6.8. *M. marinum* strains lacking the Group II (*espB, espK,* or *espJ,* teal) or Group III (*espE, espF*, purple) substrate genes were non-hemolytic following growth with tyloxapol at pH 6.8 but exhibited significantly higher hemolytic activity following growth with tyloxapol at pH 5.0. From these data, we conclude that the hemolytic activity of the WT strain following growth at pH 5.0 was independent of the Group II or Group III substrates. Moreover, we conclude that the EspA and EspC substrates can mediate hemolytic activity of *M. marinum* following growth at pH 5.0, but not at pH 6.8.

To rule out impacts of detergent or pH in PDIM (phthiocerol dimycocerosate)/PGL (phenolic glycolipid) virulence lipid levels (56, 57), which impact hemolytic activity (58, 59), we isolated total lipids from wild-type *M. marinum* grown in 7H9 media buffered to pH 6.8 or pH 5.0 with either 0.1% Tween-80 or 0.2% tyloxapol. We visualized PDIM, PGL, and TAG (triacylglycerol) using thin layer chromatography. As shown in Fig. S4, all four conditions resulted in similar levels of detectable PDIM and PGLs. Growth at pH 5.0 with 0.1% Tween-80 and in the presence of 0.2% tyloxapol at either pH resulted in reduced TAG compared to the WT strain grown at pH 6.8 with 0.1% Tween-80. From these data, we conclude that there were not gross changes to PDIM or PGL under the conditions used in this study.

We previously demonstrated that the Δ*mas M. marinum* strains lacking the PDIM and PGL virulence lipids exhibited reduced hemolytic activity and that it specifically secreted significantly reduced levels of the EspE and EspF Group III substrates (59). We demonstrated that these phenotypes could be complemented by the expression of *mas* in the Δ*mas* strain (59). We tested how the hemolytic activity of the Δ*mas* strain was impacted by growth in tween or tyloxapol, at pH 6.8 or 5.0 (Fig. 3D). As shown in Fig. 3E, the Δ*mas* strain exhibited significantly reduced hemolytic activity compared to the WT strain following growth in media buffered to pH 6.8 with 0.1% Tween-80. The Δ*mas* strain was non-hemolytic following growth in media buffered to pH 6.8 with 0.2% tyloxapol, supporting that the Group II/ Group III substrates mediate the reduced hemolytic activity in this strain. Upon shifting to growth in media buffered to pH 5.0, the Δ*mas* strain was significantly more hemolytic regardless of growth in either detergent. These data further support our findings that the substrates required for the hemolytic activity of *M. marinum* change following exposure to acidic conditions.

### *M. marinum* switches substrate secretion in response to acidic pH

We established conditions to measure ESX-1 substrate production and secretion from *M. marinum* in response to the slightly acidic (pH 6.8) or acidic (pH 5.0) conditions used in our hemolysis assays. We grew *M. marinum* in 7H9 and then diluted to an OD_600_ of 0.8 into 7H9 media buffered to pH 6.8 or pH 5.0, or Sauton’s defined media for 48 h, and tested for the secretion of the Group I substrate, EsxB. EsxB (CFP-10) is one of the canonical ESX-1 substrates used to optimize the secretion assay in Sauton’s media in the initial ESX-1 secretion papers in *M. tuberculosis, M. smegmatis,* and *M. marinum* (8, 43, 60). As shown in Fig. S5, EsxB was secreted from the WT strain under all three conditions although, to a lesser extent, from the WT strain grown in the 7H9 media buffered to either pH (lanes 1 and 2) as compared to Sauton’s media (our standard protocol, lane 3). The deletion of the *eccCb_1_* gene resulted in a loss of EsxB secretion under all conditions tested (lanes 4–6).

**Fig 4:**
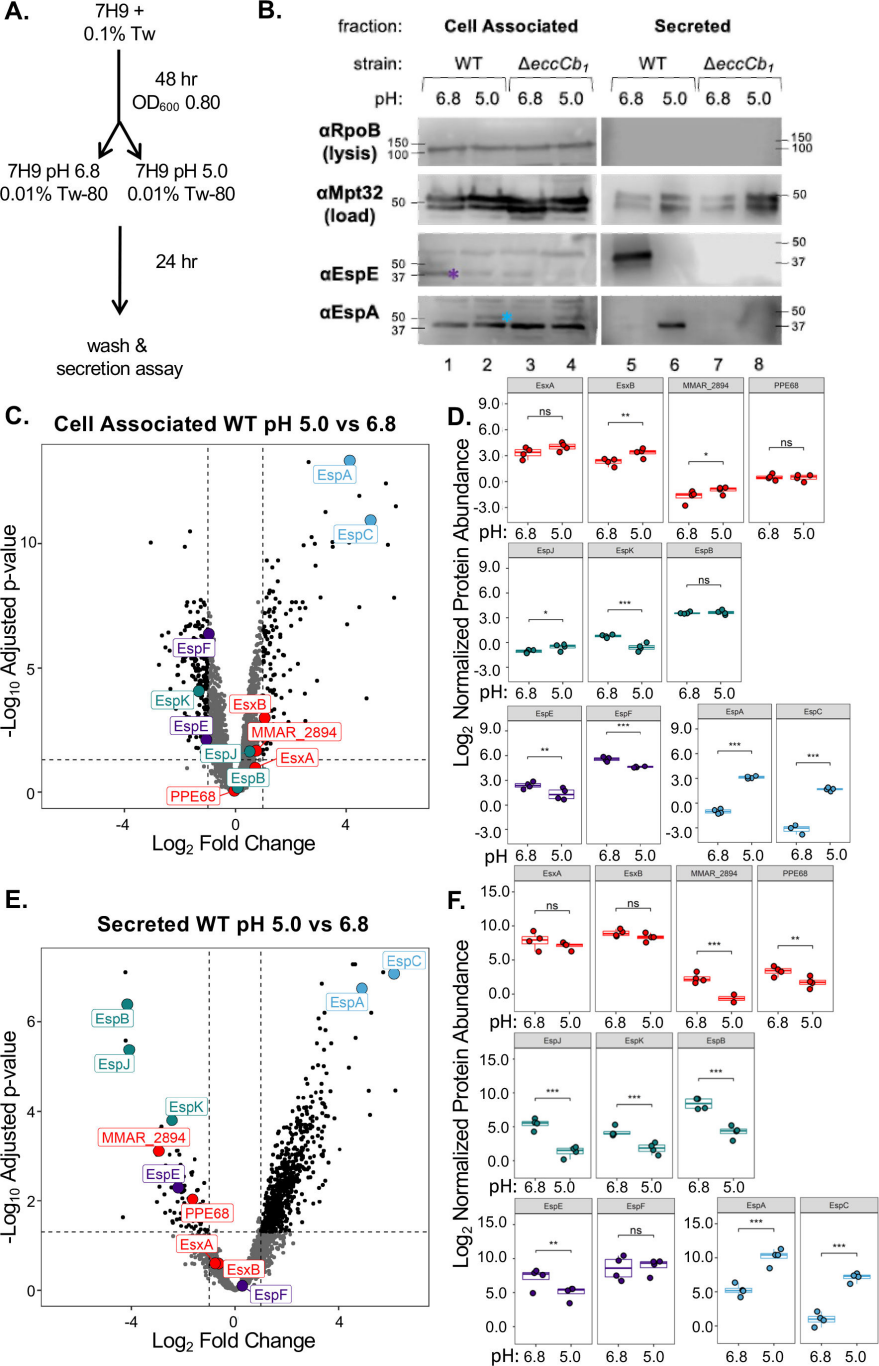
Growth at acidic pH switches ESX-1 substrate secretion. (**A**) Schematic of growth conditions for panel B. (**B**) Immunoblot of 20 µg of *M. marinum* cell-associated (left) and secreted protein fractions (right). RpoB is a control for lysis. MPT-32 is a loading control for the secreted fractions. The blot is representative of three independent biological replicates. Molecular weight markers shown in kilodaltons. The cell associated proteins were resolved on a 12% acrylamide gel. The secreted proteins were resolved on a 4%–20% min-protean TGX gel. (**C and E**) Volcano plots show log_2_ fold change (LFC) of WT strains between pH 6.8 and pH 5.0 plotted against the –log_10_ adjusted *P*-value for each protein. Dotted lines are drawn at LFC of −1 and 1, and a significance cutoff of adjusted *P*-value = 0.05. The loading controls from the western blot in panel B are replotted as a whisker plot in Fig. S7. (**D and F**) Box and whisker plots show independent biological replicates (each is an average of two technical replicates) for each protein with the significance annotation from the Benjamini-Hochberg adjusted *P*-value (ns = not significant, * = adj.*P*.value < 0.05, ** = adj.*P*.value < 0.01, *** = adj.*P*.value < 0.001) as calculated using the biological replicates (*n* = 4).

We reasoned that the differential requirements for hemolysis may reflect a switch between which ESX-1 substrate secretion in response to acidic pH. Our data in Fig. 3 suggest that both Group III (EspE/F) and Group IV (EspA/C) substrates promote hemolysis following growth at pH 6.8, while the Group IV substrates (EspA/EspC) only contribute to hemolysis following growth at pH 5.0. We hypothesized that the secretion of the Group III and Group IV substrates would switch in response to pH. We generated cell-associated and secreted protein fractions (Fig. 4A) and detected EspE and EspA production and secretion using immunoblot analysis. As shown in Fig. 4B, the EspE protein (purple asterisk) was produced in the WT and Δ*eccCb_1_* strains during growth in media buffered to pH 6.8 (lanes 1 and 3) and was secreted from the WT strain (lane 5) but not the Δ*eccCb_1_* (lane 7) strain. While EspE was detected in the cell-associated fractions of the WT and the Δ*eccCb_1_* strains grown at pH 5.0 (lanes 2 and 4), the levels of EspE were reduced. The EspE protein was not detected in the secreted protein fractions of the WT or the Δ*eccCb_1_* strain following growth at pH 5.0. In contrast, the EspA protein (light blue asterisk) was detected in the cell-associated protein fractions from the WT and Δ*eccCb_1_* strains following growth in media buffered to pH 5.0 (lanes 2 and 4), but not pH 6.8 (lanes 1 and 3). EspA was detected in the secreted fraction generated from the WT strain following growth at pH 5.0 (lane 6). From these data, we conclude that the secretion of the Group III substrate, EspE, and the Group IV substrate, EspA, switches in response to acidic pH.

To determine how growth in acidic pH alters the *M. marinum* proteome, we measured changes to protein abundance and secretion using mass spectrometry-based proteomics. Data Independent Acquisition mass spectrometry was performed (Data set S1). In the cell-associated fraction of the WT strain, we quantified 3,215 and 3,236 proteins from samples grown at pH 5.0 and pH 6.8, respectively (Fig. 4C). We measured significant changes in the levels of cell-associated proteins, with 119 significantly increased and 189 significantly decreased in pH 5.0 over pH 6.8. The levels of EspA and EspC (Group IV substrates) were increased 17- and 29-fold (or 4.12 and 4.87 log_2_ fold change, LFC) at pH 5 compared to pH 6.8. We measured smaller, but significant changes in the levels of the EspK (Group II) and EspE/EspF (Group III) substrates (Fig. 4E). Notably, we did not measure significant changes to the levels of the ESX-1 membrane complex components (EccB_1_, EccCa_1_, EccCb_1_, EccD_1_, EccE_1_, or MycP3_1, Fig. S6), supporting that the ESX-1 system is functional under both conditions. We measured significant reductions in the cell-associated levels of EccA and EspG, which are proposed to interact with and function as cytoplasmic chaperones for subsets of the ESX-1 substrates (EspF/EspC [49] and PPE68/MMAR_2894 [61, 62], respectively, Fig. S6). We also measured significant increases in the levels of EspD, which is co-transcribed with *espAC* and EspR, a transcription factor regulating the *espACD* operon (63).

In the secreted fraction, we measured 2,793 proteins and 2,170 proteins from samples grown at pH 5.0 and pH 6.8, respectively (Fig. 4E). Consistent with our prior report (34), the secretion of the ESX-1 substrates was significantly reduced in the Δ*eccCb_1_* strain compared to the WT and Δ*eccCb_1_*/p*eccCb_1_* complemented strains at pH 5.0 and pH 6.8 (Fig. S7; Data set S1). Interestingly, we measured a significant increase (30- and 71-fold or 4.90 and 6.15 LFC) in the secretion of EspA and EspC (Group IV substrates) at pH 5.0 relative to pH 6.8. We also measured significant reductions in the secretion of Group I substrates, MMAR_2894 (−2.94 LFC) and PPE68 (−1.64 LFC); the Group II substrates, EspJ (−4.09 LFC), EspB (−4.16 LFC), and EspK (−2.45 LFC); and the Group III substrate, EspE (−2.20 LFC), all of which are higher magnitude decreases in pH 5.0 than their LFC in the cell-associated fraction. Together, these data support that the abundance of the Group IV substrates and the ESX-1 chaperones are significantly changed in response to pH. Moreover, the data independently demonstrate pH-dependent substrate switching between the Group II/III and the Group IV substrates.

### Acid stress and infection models reveal changes to ESX-1 transcription and substrate requirements

**Fig 5:**
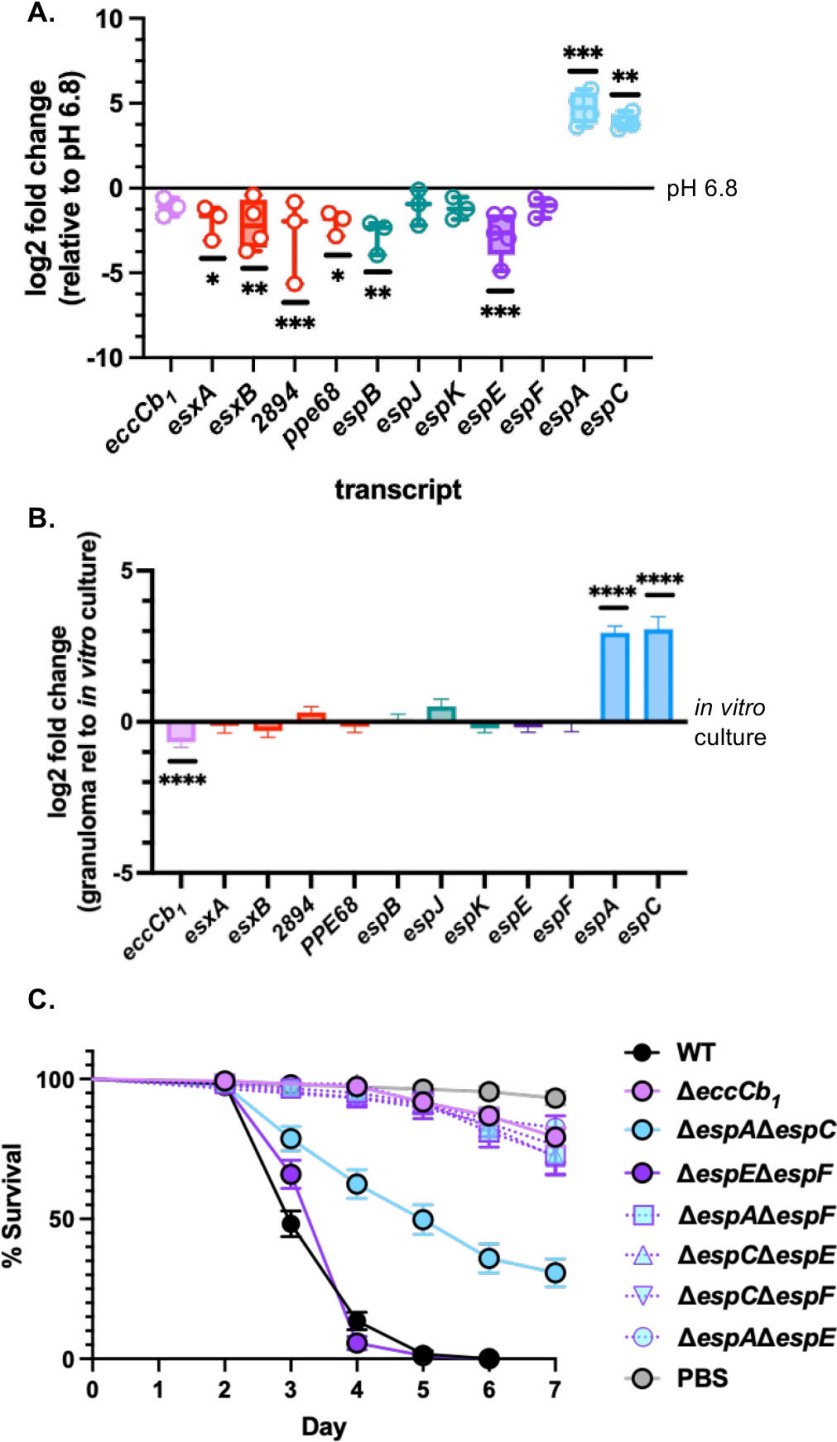
Acid stress and infection models reveal changes to ESX-1 transcription and substrate requirements during infection. (**A**) Log2 fold change of ESX-1 transcript levels in the WT strain following growth at pH 5.0 normalized to transcript levels following growth at pH 6.8 in Sauton’s media with 0.01% Tween-80. All transcripts are normalized to *sigA* levels. Each data point is a biological replicate, the average of three technical replicates. Statistical analysis was performed using one-way ordinary ANOVA (*P*< 0.0001), followed by a Dunnett’s multiple comparison test. Significance is shown relative to the same transcript measured at pH 6.8. *esxA* *P = 0.0135, *esxB* **P = 0.0036, *MMAR_2894* ***P = 0.0009, *ppe68* *P = 0.0108, *espB* **P = 0.0010, *espE* ***P = 0.0002, *espA* ***P = 0.0005, *espC* **P = 0.0083. (**B**) RNA-expression analysis of *M. marinum* genes within adult zebrafish granulomas relative to *in vitro* growth (*M. marinum* OD_600_ of ~1.0, in 7H9 supplemented with 10% OADC and 0.05% Tween-80), analyzed from RNA-seq data from (67). *n* = 4 independent biological replicates of ~400 granulomas from 8 to 12 WT zebrafish for each replicate infected with ~350 CFU for 14 days and *n* = 3 independent biological replicates for *in vitro* growth. *eccCb_1_* **** adj*P* = 0.0004, *espA* **** adj*P* = 8.32e^−36^, *espC* **** adj*P* = 5.35e^−12^. (**C**) Percent survival of *G. mellonella* infected with *M. marinum*. Larvae were injected with 10^7^ bacteria in the hindmost proleg, in 3 groups of 10 per biological replicate. Data include three biological replicates. Statistical analysis was performed using a log rank Mantel-Cox test (*P* < 0.0001). WT was not significantly different from the Δ*espE*Δ*espF* strain (*P* > 0.9999). WT was significantly different from each of the additional strains (*P* < 0.0001).

In Type III secretions systems, substrate switching is regulated through changes in substrate gene transcription (64–66). We performed relative RT-qPCR to measure the transcript levels of ESX-1 substrate genes in *M. marinum* strains grown at pH 6.8 or pH 5.0. We normalized the transcript levels following growth at pH 5.0 to the transcript levels following growth at pH 6.8 (Fig. 5A). The relative levels of the *eccCb_1_* transcript, as well as the *espJ, espK* (Group II, teal), and *espF* (Group IV, purple) transcripts were not significantly different following growth at pH 5.0, compared to growth at pH 6.8. However, the relative levels of the Group I transcripts (red), as well as *espB* (Group II, teal) and *espE* (Group IV, purple) transcripts were significantly reduced following growth at pH 5.0, compared to growth at pH 6.8. Conversely, the transcripts from the Group IV substrate genes, *espA* and *espC,* were significantly increased following growth at pH 5.0 relative to growth at pH 6.8. These data suggest that substrate switching is mediated, at least in part, by the increased transcription or transcript stability of the Group IV substrate genes during growth at acidic pH.

We next tested if ESX-1 substrate switching in response to acid stress was relevant during infection using infection models that are known to have acidic environments. Zebrafish infection with *M. marinum* readily exposes the bacteria to acidic compartments (68). We leveraged the zebrafish model of *M. marinum* infection to test if transcription of the Group III and Group IV ESX-1 substrate genes was differentially upregulated in the granuloma. As shown in Fig. 5B, the *espA* and *espC* transcripts, but not the *espE* and *espF* transcripts, were significantly upregulated in the granuloma compared to the same transcripts following *M. marinum* growth in liquid media. These data support that the Group IV transcripts are significantly increased *in vivo* in a hallmark immune structure of mycobacterial infection associated with lower pH.

Our previously published data demonstrated that in naïve macrophages, the Group III substrates are required, and the Group IV substrates are dispensable for infection by *M. marinum* (34, 44). Our hemolysis data support the hypothesis that the Group III and Group IV both contribute to virulence during acid stress. *Galleria mellonella* larvae are an established, acidic infection model for pathogenic mycobacteria, including *M. marinum* (69–71). The ESX-1 secretion system is essential for killing of *G. mellonella* by *M. marinum* (70, 72). As shown in Fig. 5C and G, *mellonella* infected with the WT *M. marinum* strain (black) had a median survival of 3 days post infection. The median survival of *G. mellonella* injected with phosphate buffered saline (PBS, gray) or infected with the ESX-1 deficient Δ*eccCb_1_* strain (light purple) had an undefined median survival because by the end of the 7-day assay, 93.20% and 79.24% of larvae survived, respectively. Similar to the larvae infected with the WT strain, larvae infected with the Δ*espE*Δ*espF M. marinum* strain, which lacks the Group III substrates, had a median survival of 4 days. The larvae infected with the Δ*espA*Δ*espC M. marinum* strain, which lacks the Group IV substrates, had a median survival of 5 days. These data suggest both the Group III and Group IV substrates can promote *G. mellonella* killing. We hypothesized that the Group IV (EspA and EspC) substrates promoted killing during infection by the Δ*espE*Δ*espF* strain, while Group III substrates (EspE and EspF) promoted killing during infection by the Δ*espA*Δ*espC* strain. To test this hypothesis, we infected *G. mellonella* with strains lacking the Group III and Group IV substrates. The *G. mellonella* infected with the Δ*espA*Δ*espE,* Δ*espF*Δ*espC*, Δ*espA*Δ*espF,* or the Δ*espE*Δ*espC M. marinum* strains had an undefined median survival, with 82.67%, 76.27%, 72.70%, and 72.10% of larvae surviving at the end of the 7-day experiment similar to the Δ*eccCb_1_* strain. Together, these data support that both the Group III and Group IV substrates contribute to virulence in an acidic infection model. Moreover, it suggests that there is likely no crosstalk between the EspE/F, EspA/C substrate pairs under these infection conditions.

## DISCUSSION

The data presented here directly support that the ESX-1 secretion system in *M. marinum* switches substrate secretion in response to acid stress, both in the laboratory and during infection. In other types of bacterial secretion systems, substrate switching occurs between secreted translocon components and from the translocon to the secreted effectors (35, 54, 65, 73). Because ESX-1 substrates are secreted in a hierarchy (34), there must be mechanisms and signals that dictate switching between the substrate groups. It remains unclear which substrates are translocon subunits and which are effectors. The Group III and Group IV substrates are encoded from paralogous loci and are dispensable for the secretion of other ESX-1-dependent substrates (34, 44), arguing against their role as part of the translocon. Moreover, our data in this study, along with our previously published data (34, 44), suggest that they cannot compensate for one another, nor is there crosstalk between the Group III and Group IV substrates under the conditions tested. One explanation consistent with our findings is that we are measuring switching between two sets of paralogous effectors that promote lysis of phagosomes or compartments of varying acidity. In this model, the Group II/Group III substrates contribute to lytic activity under both slightly acidic and acidic conditions, while the Group IV substrates only contribute to lytic activity under acidic conditions in *M. marinum*.

The roles of specific effectors of conserved secretion systems vary by species. Indeed, there are differences in the literature regarding the importance of the Group IV substrates for virulence in *M. marinum* and in *M. tuberculosis*. The deletion of the *espACD* genes attenuates *M. tuberculosis* and blocks the secretion of the EsxA and EsxB substrates under standard secretion conditions (16, 63, 74, 75). However, deletion of the *espACD* genes does not impact ESX-1 secretion under standard laboratory conditions or the virulence of *M. marinum* during macrophage infection (34). Similar to *M. marinum,* deletion of the *espEF* genes attenuates *M. tuberculosis* in infection models (76, 77). If the Group III and Group IV substrates represent paralogous effectors that function in distinct infection environments, this may explain the reported differences between the two pathogens. *M. tuberculosis* may use a different, hybrid assembly of the ESX-1 system that simultaneously requires the Group III and Group IV substrates. Alternatively, the Group IV substrates may play an expanded role in *M. tuberculosis,* while the Group III substrates have an expanded role in *M. marinum* reflecting their distinct infection niches. Further studies are underway to distinguish these possibilities.

The ESX-1 systems of both *M. marinum* and *M. tuberculosis* lyse the phagosomal membrane. Differences in these secreted effectors could explain the distinct hemolytic activities between *M. marinum* and *M. tuberculosis*. While there are some reports that *M. tuberculosis* has hemolytic activity (42)*,* there are very few compared to those reporting the robust hemolytic activity of *M. marinum*. We were able to exploit the use of *M. marinum* to demonstrate the change in substrate requirement that would not have been obvious when studying ESX-1-dependent phagosomal lysis in an asynchronous infection model. Moreover, our study demonstrates that hemolytic activity measured at pH 6.8 reflects the ability of *M. marinum* to access the macrophage cytoplasm in naïve macrophage infection and allows for the formulation of hypotheses about protein secretion that was tested and proven correct using proteomics.

Our data in *M. marinum* support that the Group II substrates, EspB, J, and K, are required for the secretion of the Group III substrates, but not the Group IV substrates. Likewise, they are dispensable for lytic activity at acidic pH. It is possible that there are additional, as of yet unidentified substrates akin to the Group II substrates that are specifically secreted under acidic conditions. These groups of substrates may serve as adaptors between the Group I substrates and the Group III/IV substrates. Although numerous studies have identified ESX substrates under standard secretion conditions, our data support that secretion may change in response to specific signals.

The Group I substrates may be both components of the translocon because they are required for the secretion of the Group II/III and IV substrates and effectors in the host. There are also paralogous loci of Group I substrates with no known function. Studies by Shah and Briken suggested that *M. tuberculosis* uses paralogous regions to act as accessory ESX-5 systems to secrete distinct subsets of substrates (78, 79). These alternate substrates, which would correspond to Group I substrates here, could be alternate ESX-5 assemblies using three paralogous loci in response to undefined signals. These studies may support that ESX systems in *M. marinum* and in *M. tuberculosis* are dynamic and tailor secretion to the environment.

A limitation of our study is that we do not yet understand the mechanistic basis for the substrate switching. Our findings indicate that transcriptional regulation likely contributes to substrate switching both during growth of *M. marinum* in the laboratory and in the zebrafish granuloma infection model. Our findings are aligned with a published data set from Pisu et al. in which they measured transcriptional signatures from *M. tuberculosis* infected mouse lung alveolar and interstitial macrophages, reflecting early intracellular infection. Our analysis of this study revealed a similar trend in the expression levels; the *espA* and *espC* transcripts, but not the *espE* and *espF* transcripts, were significantly upregulated in the lung macrophages compared to the same transcripts following *M. tuberculosis* growth in liquid media (80). We measured a significant increase in EspR levels following growth at pH 5.0. EspR positively regulates the *espACD* operon in *M. tuberculosis* (63). EspR is regulated by PhoPR, which is a pH responsive two-component system (81). Increased levels of EspA and EspC in the cytoplasm could drive increased secretion following growth under acidic conditions, as supported by our proteomic analyses. There are similar transcriptional connections in substrate switching in Type III secretion systems. However, we think this is unlikely because the *espE* and *espF* transcripts remain high in the zebrafish granuloma. Our prior proteomic analyses suggest competition between the Group II/III substrates and Group IV substrates for secretion *in vitro* (34). The deletion of individual Group II substrate genes, *espB*, *espJ,* or *espK*, resulted in significantly increased secretion of the Group IV substrates, EspA or EspC, as measured by proteomics. This finding is consistent with the proteomics data in this study. Indeed, acid stress led to a significant reduction in the secretion of the Group II substrates and a significant increase in the Group IV substrates.

In Type III secretion, competition for chaperones or machinery (sorting complex) drives substrate switching in response to environmental signals. The ESX-1 system has two chaperones, EccA and EspG. EspG directly interacts with PE/PPE proteins including PPE68 (61, 62, 82). We previously showed that both EspC and EspF directly interact with the same cytoplasmic ESX-1 component, EccA (49), supporting a potential mechanism for competition. EccA may be part of a sorting complex in the cytoplasm that dictates the order of substrate delivery to the secretion machinery. Interestingly, our proteomics analysis suggested that following exposure to acid stress, the levels of EccA were significantly reduced. It is possible that reduced EccA, with increased EspC/EspA, could result in an increase in Group IV secretion and a corresponding decrease in Group III secretion. We also found a significant reduction in EspG levels following acid stress. EspG interacts with PPE68. Reduced EspG could explain the reduction in PPE68/MMAR_2894 secretion measured following growth at pH 5.0. We are continuing to define the mechanisms underlying the initial report of substrate switching observed here.

We previously demonstrated that EspE is a negative regulator of ESX-1 gene expression through the WhiB6 transcription factor (44). While this could impact the interpretation of the double deletion strains with Δ*espE*, we do not think this is an issue. First, in our prior work, we did not measure any feedback control of the *espACD* locus by the ESX-1 system (48). Another limitation of this study is that we did not complement the double deletion strains by expressing individual ESX-1 genes. However, the major finding with the double deletion strains was that strains lacking Group III and Group IV substrates were non-hemolytic under all conditions tested. We recapitulated this finding using the Δ*espA* and the Δ*espC* deletion strains and chemical inhibition of the Group II/III ESX-1 assembly using tyloxapol. We previously showed that the growth of *M. marinum* in tyloxapol specifically blocks the hemolytic activity of the Group II/III ESX-1 assembly, significantly reducing the secretion of EspB/EspJ, EspE, and increasing the secretion of EspA/EspC (55). Disruption of the secretion of the Group II/ Group III substrates by tyloxapol in the *espA* and *espC* strains resulted in a loss of hemolytic activity under both pH conditions, and this was complemented by the expression of *espA* or *espC* accordingly.

Mycobacteria encounter environments of varying acidity during infection. In this study, we used two known acidic models of infection, *G. mellonella* and the zebrafish granuloma. However, testing this in an activated macrophage model of infection and further studies in the zebrafish model of infection will be important. Up to 90% of cells infected during mycobacterial infection are macrophages (83). The infected macrophage populations are functionally heterogeneous, controlling or promoting mycobacterial growth (83, 84). Phagosome physiology is likewise heterogeneous. When infecting naïve, resting macrophages, pathogenic mycobacteria arrest phagosome maturation, resulting in a mildly acidic phagosome (pH ~6.2). However, in activated macrophages, the lysosome fuses with the mycobacteria-containing phagosome, resulting in an acidic phagolysosome (pH 4.5–5) (85–90). Later during infection, mycobacteria are contained within heterogeneous granulomas with a median pH of 5.5 (91, 92). Our findings may inform how pathogenic mycobacteria lyse phagosomes of differing pH and suggest that pathogenic mycobacteria have the potential to change the virulence factors that mediate phagosomal escape in response to their environment.

## MATERIALS AND METHODS

Bacterial strains were derived from the *M. marinum* M strain (ATCC BAA-535). Strains were maintained in 7H9 media and exposed to acid stress using 7H9 buffered with 3-(*N*-morpholino)propanesulfonic acid (MOPS) to either pH 5.0 or pH 6.8 with 0.1% Tween-80 or 0.2% tyloxapol as indicated. *M. marinum* deletion strains were generated using allelic exchange as (34). Hemolytic activity was measured similar to references 34, 46, except that strains were grown in buffered and/or detergent-treated media for 24 h and washed prior to hemolysis assay. RNA extraction and RT-qPCR were performed as reference 93. Protein secretion assays were performed following 48 h of growth in MOPs buffered 7H9 media (pH 5.0 or pH 6.8) with 0.01% Tween-80 with 0.5% glycerol, but without glucose. Cell associated and secreted protein fractions were generated as in reference 46. Mass Spectrometry and data analysis were performed as reference 94. The protein fractions were precipitated with acetone (95) followed by SDS-PAGE and immunoblot analysis (46). Wax worm infections were performed as reference 72. An expanded methods section is available in the Supplemental material.

## Data Availability

Raw data files, search parameters, and instrument method parameters were deposited in massIVE (identifier MSV000100192) and Proteome Xchange (identifier PXD071871).

## SUPPLEMENTAL MATERIAL

Data set S1 msphere.00056-26-s0001.xlsb Proteomics data set.

Supplemental Material msphere.00056-26-s0002.pdf Supplemental figures, methods, and tables.

ASM does not own the copyrights to Supplemental Material that may be linked to, or accessed through, an article. The authors have granted ASM a non-exclusive, world-wide license to publish the Supplemental Material files. Please contact the corresponding author directly for reuse.

## References

[1] Suter E. 1952. The multiplication of tubercle bacilli within normal phagocytes in tissue culture. J Exp Med 96:137–150. doi:10.1084/jem.96.2.137 14955570 PMC2136137

[2] Armstrong JA, Hart PD. 1971. Response of cultured macrophages to Mycobacterium tuberculosis, with observations on fusion of lysosomes with phagosomes. J Exp Med 134:713–740. doi:10.1084/jem.134.3.713 15776571 PMC2139093

[3] Armstrong JA, Hart PD. 1975. Phagosome-lysosome interactions in cultured macrophages infected with virulent tubercle bacilli. Reversal of the usual nonfusion pattern and observations on bacterial survival. J Exp Med 142:1–16. doi:10.1084/jem.142.1.1 807671 PMC2189870

[4] Rohde KH, Abramovitch RB, Russell DG. 2007. Mycobacterium tuberculosis invasion of macrophages: linking bacterial gene expression to environmental cues. Cell Host Microbe 2:352–364. doi:10.1016/j.chom.2007.09.006 18005756

[5] Gouzy A, Healy C, Black KA, Rhee KY, Ehrt S. 2021. Growth of Mycobacterium tuberculosis at acidic pH depends on lipid assimilation and is accompanied by reduced GAPDH activity. Proc Natl Acad Sci USA 118:e2024571118. doi:10.1073/pnas.2024571118 34341117 PMC8364206

[6] Russell DG. 2001. Mycobacterium tuberculosis: here today, and here tomorrow. Nat Rev Mol Cell Biol 2:569–577. doi:10.1038/35085034 11483990

[7] Ehrt S, Schnappinger D. 2009. Mycobacterial survival strategies in the phagosome: defence against host stresses. Cell Microbiol 11:1170–1178. doi:10.1111/j.1462-5822.2009.01335.x 19438516 PMC3170014

[8] Stanley SA, Raghavan S, Hwang WW, Cox JS. 2003. Acute infection and macrophage subversion by Mycobacterium tuberculosis require a specialized secretion system. Proc Natl Acad Sci USA 100:13001–13006. doi:10.1073/pnas.2235593100 14557536 PMC240734

[9] van der Wel N, Hava D, Houben D, Fluitsma D, van Zon M, Pierson J, Brenner M, Peters PJ. 2007. M. tuberculosis and M. leprae translocate from the phagolysosome to the cytosol in myeloid cells. Cell 129:1287–1298. doi:10.1016/j.cell.2007.05.059 17604718

[10] Watson RO, Manzanillo PS, Cox JS. 2012. Extracellular M. tuberculosis DNA targets bacteria for autophagy by activating the host DNA-sensing pathway. Cell 150:803–815. doi:10.1016/j.cell.2012.06.040 22901810 PMC3708656

[11] Houben D, Demangel C, van Ingen J, Perez J, Baldeón L, Abdallah AM, Caleechurn L, Bottai D, van Zon M, de Punder K, van der Laan T, Kant A, Bossers-de Vries R, Willemsen P, Bitter W, van Soolingen D, Brosch R, van der Wel N, Peters PJ. 2012. ESX-1-mediated translocation to the cytosol controls virulence of mycobacteria. Cell Microbiol 14:1287–1298. doi:10.1111/j.1462-5822.2012.01799.x 22524898

[12] Ramakrishnan L, Federspiel NA, Falkow S. 2000. Granuloma-specific expression of Mycobacterium virulence proteins from the glycine-rich PE-PGRS family. Science 288:1436–1439. doi:10.1126/science.288.5470.1436 10827956

[13] McLaughlin B, Chon JS, MacGurn JA, Carlsson F, Cheng TL, Cox JS, Brown EJ. 2007. A mycobacterium ESX-1-secreted virulence factor with unique requirements for export. PLoS Pathog 3:e105. doi:10.1371/journal.ppat.0030105 17676952 PMC1937011

[14] Carlsson F, Joshi SA, Rangell L, Brown EJ. 2009. Polar localization of virulence-related Esx-1 secretion in mycobacteria. PLoS Pathog 5:e1000285. doi:10.1371/journal.ppat.1000285 19180234 PMC2628743

[15] Champion MM, Williams EA, Pinapati RS, Champion PAD. 2014. Correlation of phenotypic profiles using targeted proteomics identifies mycobacterial Esx-1 substrates. J Proteome Res 13:5151–5164. doi:10.1021/pr500484w 25106450 PMC4227905

[16] Fortune SM, Jaeger A, Sarracino DA, Chase MR, Sassetti CM, Sherman DR, Bloom BR, Rubin EJ. 2005. Mutually dependent secretion of proteins required for mycobacterial virulence. Proc Natl Acad Sci USA 102:10676–10681. doi:10.1073/pnas.0504922102 16030141 PMC1176248

[17] Bosserman RE, Nicholson KR, Champion MM, Champion PA. 2019. A new ESX-1 substrate in Mycobacterium marinum that is required for hemolysis but not host cell lysis. J Bacteriol 201:e00760-18. doi:10.1128/JB.00760-18 30833360 PMC6597391

[18] Sayes F, Blanc C, Ates LS, Deboosere N, Orgeur M, Le Chevalier F, Gröschel MI, Frigui W, Song O-R, Lo-Man R, Brossier F, Sougakoff W, Bottai D, Brodin P, Charneau P, Brosch R, Majlessi L. 2018. Multiplexed quantitation of intraphagocyte Mycobacterium tuberculosis secreted protein effectors. Cell Rep 23:1072–1084. doi:10.1016/j.celrep.2018.03.125 29694886 PMC5946722

[19] Leddy O, White FM, Bryson BD. 2023. Immunopeptidomics reveals determinants of Mycobacterium tuberculosis antigen presentation on MHC class I. eLife 12:e84070. doi:10.7554/eLife.84070 37073954 PMC10159623

[20] Quigley J, Hughitt VK, Velikovsky CA, Mariuzza RA, El-Sayed NM, Briken V. 2017. The cell wall lipid PDIM contributes to phagosomal escape and host cell exit of Mycobacterium tuberculosis. mBio 8:e00148-17. doi:10.1128/mBio.00148-17 28270579 PMC5340868

[21] Barczak AK, Avraham R, Singh S, Luo SS, Zhang WR, Bray M-A, Hinman AE, Thompson M, Nietupski RM, Golas A, Montgomery P, Fitzgerald M, Smith RS, White DW, Tischler AD, Carpenter AE, Hung DT. 2017. Systematic, multiparametric analysis of Mycobacterium tuberculosis intracellular infection offers insight into coordinated virulence. PLoS Pathog 13:e1006363. doi:10.1371/journal.ppat.1006363 28505176 PMC5444860

[22] Augenstreich J, Briken V. 2020. Host cell targets of released lipid and secreted protein effectors of Mycobacterium tuberculosis. Front Cell Infect Microbiol 10:595029. doi:10.3389/fcimb.2020.595029 33194845 PMC7644814

[23] Stanley SA, Johndrow JE, Manzanillo P, Cox JS. 2007. The type I IFN response to infection with Mycobacterium tuberculosis requires ESX-1-mediated secretion and contributes to pathogenesis. J Immunol 178:3143–3152. doi:10.4049/jimmunol.178.5.3143 17312162

[24] Wassermann R, Gulen MF, Sala C, Perin SG, Lou Y, Rybniker J, Schmid-Burgk JL, Schmidt T, Hornung V, Cole ST, Ablasser A. 2015. Mycobacterium tuberculosis differentially activates cGAS- and inflammasome-dependent intracellular immune responses through ESX-1. Cell Host Microbe 17:799–810. doi:10.1016/j.chom.2015.05.003 26048138

[25] Lienard J, Nobs E, Lovins V, Movert E, Valfridsson C, Carlsson F. 2020. The Mycobacterium marinum ESX-1 system mediates phagosomal permeabilization and type I interferon production via separable mechanisms. Proc Natl Acad Sci USA 117:1160–1166. doi:10.1073/pnas.1911646117 31879349 PMC6969537

[26] Pandey AK, Yang Y, Jiang Z, Fortune SM, Coulombe F, Behr MA, Fitzgerald KA, Sassetti CM, Kelliher MA. 2009. NOD2, RIP2 and IRF5 play a critical role in the type I interferon response to Mycobacterium tuberculosis. PLoS Pathog 5:e1000500. doi:10.1371/journal.ppat.1000500 19578435 PMC2698121

[27] Lee AM, Nathan CF. 2024. Type I interferon exacerbates Mycobacterium tuberculosis induced human macrophage death. EMBO Rep 25:3064–3089. doi:10.1038/s44319-024-00171-0 38866980 PMC11239827

[28] Linell F, Norden A. 1954. Mycobacterium balnei, a new acid-fast bacillus occurring in swimming pools and capable of producing skin lesions in humans. Acta Tuberc Scand Suppl 33:1–84. 13188762

[29] Stamm LM, Brown EJ. 2004. Mycobacterium marinum: the generalization and specialization of a pathogenic mycobacterium. Microbes Infect 6:1418–1428. doi:10.1016/j.micinf.2004.10.003 15596129

[30] Pozos TC, Ramakrishnan L. 2004. New models for the study of Mycobacterium-host interactions. Curr Opin Immunol 16:499–505. doi:10.1016/j.coi.2004.05.011 15245746

[31] Menon AR, Prest RJ, Tobin DM, Champion PA. 2025. Mycobacterium marinum as a model for understanding principles of mycobacterial pathogenesis. J Bacteriol 207:e00047-25. doi:10.1128/jb.00047-25 40304497 PMC12096832

[32] Tobin DM, Ramakrishnan L. 2008. Comparative pathogenesis of Mycobacterium marinum and Mycobacterium tuberculosis. Cell Microbiol 10:1027–1039. doi:10.1111/j.1462-5822.2008.01133.x 18298637

[33] Stinear TP, Seemann T, Harrison PF, Jenkin GA, Davies JK, Johnson PDR, Abdellah Z, Arrowsmith C, Chillingworth T, Churcher C, et al.. 2008. Insights from the complete genome sequence of Mycobacterium marinum on the evolution of Mycobacterium tuberculosis. Genome Res 18:729–741. doi:10.1101/gr.075069.107 18403782 PMC2336800

[34] Cronin RM, Ferrell MJ, Cahir CW, Champion MM, Champion PA. 2022. Proteo-genetic analysis reveals clear hierarchy of ESX-1 secretion in Mycobacterium marinum. Proc Natl Acad Sci USA 119:e2123100119. doi:10.1073/pnas.2123100119 35671426 PMC9214503

[35] Deane JE, Abrusci P, Johnson S, Lea SM. 2010. Timing is everything: the regulation of type III secretion. Cell Mol Life Sci 67:1065–1075. doi:10.1007/s00018-009-0230-0 20043184 PMC2835726

[36] Piton J, Pojer F, Wakatsuki S, Gati C, Cole ST. 2020. High resolution CryoEM structure of the ring-shaped virulence factor EspB from Mycobacterium tuberculosis. J Struct Biol X 4:100029. doi:10.1016/j.yjsbx.2020.100029 32875288 PMC7451430

[37] Wagner JM, Chan S, Evans TJ, Kahng S, Kim J, Arbing MA, Eisenberg D, Korotkov KV. 2016. Structures of EccB1 and EccD1 from the core complex of the mycobacterial ESX-1 type VII secretion system. BMC Struct Biol 16:5. doi:10.1186/s12900-016-0056-6 26922638 PMC4769845

[38] Beckham KSH, Ciccarelli L, Bunduc CM, Mertens HDT, Ummels R, Lugmayr W, Mayr J, Rettel M, Savitski MM, Svergun DI, Bitter W, Wilmanns M, Marlovits TC, Parret AHA, Houben ENG. 2017. Structure of the mycobacterial ESX-5 type VII secretion system membrane complex by single-particle analysis. Nat Microbiol 2:17047. doi:10.1038/nmicrobiol.2017.47 28394313

[39] Poweleit N, Czudnochowski N, Nakagawa R, Trinidad DD, Murphy KC, Sassetti CM, Rosenberg OS. 2019. The structure of the endogenous ESX-3 secretion system. eLife 8:e52983. doi:10.7554/eLife.52983 31886769 PMC6986878

[40] Famelis N, Rivera-Calzada A, Degliesposti G, Wingender M, Mietrach N, Skehel JM, Fernandez-Leiro R, Böttcher B, Schlosser A, Llorca O, Geibel S. 2019. Architecture of the mycobacterial type VII secretion system. Nature 576:321–325. doi:10.1038/s41586-019-1633-1 31597161 PMC6914368

[41] Guinn KM, Hickey MJ, Mathur SK, Zakel KL, Grotzke JE, Lewinsohn DM, Smith S, Sherman DR. 2004. Individual RD1-region genes are required for export of ESAT-6/CFP-10 and for virulence of Mycobacterium tuberculosis. Mol Microbiol 51:359–370. doi:10.1046/j.1365-2958.2003.03844.x 14756778 PMC1458497

[42] King CH, Mundayoor S, Crawford JT, Shinnick TM. 1993. Expression of contact-dependent cytolytic activity by Mycobacterium tuberculosis and isolation of the genomic locus that encodes the activity. Infect Immun 61:2708–2712. doi:10.1128/iai.61.6.2708-2712.1993 8500911 PMC280905

[43] Gao LY, Guo S, McLaughlin B, Morisaki H, Engel JN, Brown EJ. 2004. A mycobacterial virulence gene cluster extending RD1 is required for cytolysis, bacterial spreading and ESAT-6 secretion. Mol Microbiol 53:1677–1693. doi:10.1111/j.1365-2958.2004.04261.x 15341647

[44] Chirakos AE, Nicholson KR, Huffman A, Champion PA. 2020. Conserved ESX-1 substrates EspE and EspF are virulence factors that regulate gene expression. Infect Immun 88:e00289-20. doi:10.1128/IAI.00289-20 32900815 PMC7671884

[45] Sauer JD, Witte CE, Zemansky J, Hanson B, Lauer P, Portnoy DA. 2010. Listeria monocytogenes triggers AIM2-mediated pyroptosis upon infrequent bacteriolysis in the macrophage cytosol. Cell Host Microbe 7:412–419. doi:10.1016/j.chom.2010.04.004 20417169 PMC2947455

[46] Collars OA, Jones BS, Hu DD, Weaver SD, Sherman TA, Champion MM, Champion PA. 2023. An N-acetyltransferase required for ESAT-6 N-terminal acetylation and virulence in Mycobacterium marinum. mBio 14:e00987-23. doi:10.1128/mbio.00987-23 37772840 PMC10653941

[47] Manzanillo PS, Shiloh MU, Portnoy DA, Cox JS. 2012. Mycobacterium tuberculosis activates the DNA-dependent cytosolic surveillance pathway within macrophages. Cell Host Microbe 11:469–480. doi:10.1016/j.chom.2012.03.007 22607800 PMC3662372

[48] Bosserman RE, Nguyen TT, Sanchez KG, Chirakos AE, Ferrell MJ, Thompson CR, Champion MM, Abramovitch RB, Champion PA. 2017. WhiB6 regulation of ESX-1 gene expression is controlled by a negative feedback loop in Mycobacterium marinum. Proc Natl Acad Sci USA 114:E10772–E10781. doi:10.1073/pnas.1710167114 29180415 PMC5740670

[49] Champion PAD, Champion MM, Manzanillo P, Cox JS. 2009. ESX-1 secreted virulence factors are recognized by multiple cytosolic AAA ATPases in pathogenic mycobacteria. Mol Microbiol 73:950–962. doi:10.1111/j.1365-2958.2009.06821.x 19682254 PMC3023814

[50] Buttner D. 2012. Protein export according to schedule: architecture, assembly, and regulation of type III secretion systems from plant- and animal-pathogenic bacteria. Microbiol Mol Biol Rev 76:262–310. doi:10.1128/MMBR.05017-11 22688814 PMC3372255

[51] Dewoody RS, Merritt PM, Marketon MM. 2013. Regulation of the Yersinia type III secretion system: traffic control. Front Cell Infect Microbiol 3:4. doi:10.3389/fcimb.2013.00004 23390616 PMC3565153

[52] Boll JM, Hendrixson DR. 2013. A regulatory checkpoint during flagellar biogenesis in Campylobacter jejuni initiates signal transduction to activate transcription of flagellar genes. mBio 4:e00432-13. doi:10.1128/mBio.00432-13 24003178 PMC3760246

[53] Notti RQ, Stebbins CE. 2016. The structure and function of type III secretion systems. Microbiol Spectr 4. doi:10.1128/microbiolspec.VMBF-0004-2015 PMC4804468 26999392

[54] Muthuramalingam M, Whittier SK, Picking WL, Picking WD. 2021. The Shigella type III secretion system: an overview from top to bottom. Microorganisms 9:451. doi:10.3390/microorganisms9020451 33671545 PMC7926512

[55] Collars OA, Weaver SD, Hernandez RL, Champion MM, Champion PA. 2026. Tyloxapol inhibits ESX-1 secretion in Mycobacterium marinum. J Bacteriol:e0013626. doi:10.1128/jb.00136-26 42017691 PMC13192266

[56] Cox JS, Chen B, McNeil M, Jacobs WR Jr. 1999. Complex lipid determines tissue-specific replication of Mycobacterium tuberculosis in mice. Nature 402:79–83. doi:10.1038/47042 10573420

[57] Yu J, Tran V, Li M, Huang X, Niu C, Wang D, Zhu J, Wang J, Gao Q, Liu J. 2012. Both phthiocerol dimycocerosates and phenolic glycolipids are required for virulence of Mycobacterium marinum. Infect Immun 80:1381–1389. doi:10.1128/IAI.06370-11 22290144 PMC3318414

[58] Osman MM, Pagán AJ, Shanahan JK, Ramakrishnan L. 2020. Mycobacterium marinum phthiocerol dimycocerosates enhance macrophage phagosomal permeabilization and membrane damage. PLoS One 15:e0233252. doi:10.1371/journal.pone.0233252 32701962 PMC7377490

[59] Jones BS, Hu DD, Nicholson KR, Cronin RM, Weaver SD, Champion MM, Champion PA. 2024. The loss of the PDIM/PGL virulence lipids causes differential secretion of ESX-1 substrates in Mycobacterium marinum. mSphere 9:e00005-24. doi:10.1128/msphere.00005-24 38661343 PMC11237470

[60] Converse SE, Cox JS. 2005. A protein secretion pathway critical for Mycobacterium tuberculosis virulence is conserved and functional in Mycobacterium smegmatis. J Bacteriol 187:1238–1245. doi:10.1128/JB.187.4.1238-1245.2005 15687187 PMC545616

[61] Damen MPM, Phan TH, Ummels R, Rubio-Canalejas A, Bitter W, Houben ENG. 2020. Modification of a PE/PPE substrate pair reroutes an ESX substrate pair from the mycobacterial ESX-1 type VII secretion system to the ESX-5 system. J Biol Chem 295:5960–5969. doi:10.1074/jbc.RA119.011682 32184351 PMC7196631

[62] Damen MPM, Meijers AS, Keizer EM, Piersma SR, Jiménez CR, Kuijl CP, Bitter W, Houben ENG. 2022. The ESX-1 substrate PPE68 has a key function in ESX-1-mediated secretion in Mycobacterium marinum. mBio 13:e02819-22. doi:10.1128/mbio.02819-22 36409073 PMC9765416

[63] Raghavan S, Manzanillo P, Chan K, Dovey C, Cox JS. 2008. Secreted transcription factor controls Mycobacterium tuberculosis virulence. Nature 454:717–721. doi:10.1038/nature07219 18685700 PMC2862998

[64] Parsot C, Ageron E, Penno C, Mavris M, Jamoussi K, d’Hauteville H, Sansonetti P, Demers B. 2005. A secreted anti-activator, OspD1, and its chaperone, Spa15, are involved in the control of transcription by the type III secretion apparatus activity in Shigella flexneri. Mol Microbiol 56:1627–1635. doi:10.1111/j.1365-2958.2005.04645.x 15916611

[65] Portaliou AG, Tsolis KC, Loos MS, Balabanidou V, Rayo J, Tsirigotaki A, Crepin VF, Frankel G, Kalodimos CG, Karamanou S, Economou A. 2017. Hierarchical protein targeting and secretion is controlled by an affinity switch in the type III secretion system of enteropathogenic Escherichia coli. EMBO J 36:3517–3531. doi:10.15252/embj.201797515 29109154 PMC5709732

[66] Du Z, Tan Y, Yang H, Qiu J, Qin L, Wang T, Liu H, Bi Y, Song Y, Guo Z, Han Y, Zhou D, Wang X, Yang R. 2009. Gene expression profiling of Yersinia pestis with deletion of lcrG, a known negative regulator for Yop secretion of type III secretion system. Int J Med Microbiol 299:355–366. doi:10.1016/j.ijmm.2008.10.003 19109068

[67] Viswanathan G, Hughes EJ, Gan M, Xet-Mull AM, Lowy JP, Pyle CJ, Alexander G, Swain-Lenz D, Liu Q, Tobin DM. 2026. Granuloma dual RNA-seq reveals composite transcriptional programs driven by neutrophils and necrosis within tuberculous granulomas. Sci Adv 12:eadw4619. doi:10.1126/sciadv.adw4619 41564181 PMC12822655

[68] Levitte S, Adams KN, Berg RD, Cosma CL, Urdahl KB, Ramakrishnan L. 2016. Mycobacterial acid tolerance enables phagolysosomal survival and establishment of tuberculous infection in vivo. Cell Host Microbe 20:250–258. doi:10.1016/j.chom.2016.07.007 27512905 PMC4985559

[69] Li Y, Spiropoulos J, Cooley W, Khara JS, Gladstone CA, Asai M, Bossé JT, Robertson BD, Newton SM, Langford PR. 2018. Galleria mellonella - a novel infection model for the Mycobacterium tuberculosis complex. Virulence 9:1126–1137. doi:10.1080/21505594.2018.1491255 30067135 PMC6086298

[70] Asai M, Li Y, Spiropoulos J, Cooley W, Everest DJ, Kendall SL, Martín C, Robertson BD, Langford PR, Newton SM. 2022. Galleria mellonella as an infection model for the virulent Mycobacterium tuberculosis H37Rv. Virulence 13:1543–1557. doi:10.1080/21505594.2022.2119657 36052440 PMC9481108

[71] Campbell JS, Pearce JC, Bebes A, Pradhan A, Yuecel R, Brown AJP, Wakefield JG. 2024. Characterising phagocytes and measuring phagocytosis from live Galleria mellonella larvae. Virulence 15:2313413. doi:10.1080/21505594.2024.2313413 38357909 PMC10877982

[72] Jones BS, Pareek V, Hu DD, Weaver SD, Syska C, Galfano G, Champion MM, Champion PA. 2025. N-acetyltransferases required for iron uptake and aminoglycoside resistance promote virulence lipid production in Mycobacterium marinum. Proc Natl Acad Sci USA 122:e2502577122. doi:10.1073/pnas.2502577122 40680026 PMC12305045

[73] Williams McMackin EA, Djapgne L, Corley JM, Yahr TL. 2019. Fitting pieces into the puzzle of Pseudomonas aeruginosa type III secretion system gene expression. J Bacteriol 201:e00209-19. doi:10.1128/JB.00209-19 31010903 PMC6560140

[74] MacGurn JA, Raghavan S, Stanley SA, Cox JS. 2005. A non-RD1 gene cluster is required for Snm secretion in Mycobacterium tuberculosis. Mol Microbiol 57:1653–1663. doi:10.1111/j.1365-2958.2005.04800.x 16135231

[75] Johnson BK, Colvin CJ, Needle DB, Mba Medie F, Champion PAD, Abramovitch RB. 2015. The carbonic anhydrase inhibitor ethoxzolamide inhibits the Mycobacterium tuberculosis PhoPR regulon and Esx-1 secretion and attenuates virulence. Antimicrob Agents Chemother 59:4436–4445. doi:10.1128/AAC.00719-15 25987613 PMC4505220

[76] Bottai D, Majlessi L, Simeone R, Frigui W, Laurent C, Lenormand P, Chen J, Rosenkrands I, Huerre M, Leclerc C, Cole ST, Brosch R. 2011. ESAT-6 secretion-independent impact of ESX-1 genes espF and espG_1_ on virulence of Mycobacterium tuberculosis. J Infect Dis 203:1155–1164. doi:10.1093/infdis/jiq089 21196469

[77] Brodin P, Majlessi L, Marsollier L, de Jonge MI, Bottai D, Demangel C, Hinds J, Neyrolles O, Butcher PD, Leclerc C, Cole ST, Brosch R. 2006. Dissection of ESAT-6 system 1 of Mycobacterium tuberculosis and impact on immunogenicity and virulence. Infect Immun 74:88–98. doi:10.1128/IAI.74.1.88-98.2006 16368961 PMC1346617

[78] Shah S, Cannon JR, Fenselau C, Briken V. 2015. A duplicated ESAT-6 region of ESX-5 is involved in protein export and virulence of mycobacteria. Infect Immun 83:4349–4361. doi:10.1128/IAI.00827-15 26303392 PMC4598393

[79] Shah S, Briken V. 2016. Modular organization of the ESX-5 secretion system in Mycobacterium tuberculosis. Front Cell Infect Microbiol 6:49. doi:10.3389/fcimb.2016.00049 27200304 PMC4852179

[80] Pisu D, Huang L, Grenier JK, Russell DG. 2020. Dual RNA-Seq of Mtb-infected macrophages in vivo reveals ontologically distinct host-pathogen interactions. Cell Rep 30:335–350. doi:10.1016/j.celrep.2019.12.033 31940480 PMC7032562

[81] Anil Kumar V, Goyal R, Bansal R, Singh N, Sevalkar RR, Kumar A, Sarkar D. 2016. EspR-dependent ESAT-6 protein secretion of Mycobacterium tuberculosis requires the presence of virulence regulator PhoP. J Biol Chem 291:19018–19030. doi:10.1074/jbc.M116.746289 27445330 PMC5009273

[82] Phan TH, Ummels R, Bitter W, Houben ENG. 2017. Identification of a substrate domain that determines system specificity in mycobacterial type VII secretion systems. Sci Rep 7:42704. doi:10.1038/srep42704 28205541 PMC5311947

[83] Pisu D, Huang L, Narang V, Theriault M, Lê-Bury G, Lee B, Lakudzala AE, Mzinza DT, Mhango DV, Mitini-Nkhoma SC, Jambo KC, Singhal A, Mwandumba HC, Russell DG. 2021. Single cell analysis of M. tuberculosis phenotype and macrophage lineages in the infected lung. J Exp Med 218:e20210615. doi:10.1084/jem.20210615 34292313 PMC8302446

[84] Russell DG, Huang L, VanderVen BC. 2019. Immunometabolism at the interface between macrophages and pathogens. Nat Rev Immunol 19:291–304. doi:10.1038/s41577-019-0124-9 30679807 PMC7032560

[85] Vandal OH, Nathan CF, Ehrt S. 2009. Acid resistance in Mycobacterium tuberculosis. J Bacteriol 191:4714–4721. doi:10.1128/JB.00305-09 19465648 PMC2715723

[86] VanderVen BC, Huang L, Rohde KH, Russell DG. 2016. The minimal unit of infection: Mycobacterium tuberculosis in the macrophage. Microbiol Spectr 4. doi:10.1128/microbiolspec.TBTB2-0025-2016 PMC5245711 28084213

[87] MacMicking JD, Taylor GA, McKinney JD. 2003. Immune control of tuberculosis by IFN-γ-inducible LRG-47. Science 302:654–659. doi:10.1126/science.1088063 14576437

[88] Schaible UE, Sturgill-Koszycki S, Schlesinger PH, Russell DG. 1998. Cytokine activation leads to acidification and increases maturation of Mycobacterium avium-containing phagosomes in murine macrophages. J Immunol 160:1290–1296. doi:10.4049/jimmunol.160.3.1290 9570546

[89] Via LE, Fratti RA, McFalone M, Pagan-Ramos E, Deretic D, Deretic V. 1998. Effects of cytokines on mycobacterial phagosome maturation. J Cell Sci 111:897–905. doi:10.1242/jcs.111.7.897 9490634

[90] Sprick MG. 1956. Phagocytosis of M. tuberculosis and M. smegmatis stained with indicator dyes. Am Rev Tuberc 74:552–565. doi:10.1164/artpd.1956.74.4.552 13362862

[91] Ehlers S, Schaible UE. 2012. The granuloma in tuberculosis: dynamics of a host-pathogen collusion. Front Immunol 3:411. doi:10.3389/fimmu.2012.00411 23308075 PMC3538277

[92] Baker JJ, Dechow SJ, Abramovitch RB. 2019. Acid fasting: modulation of Mycobacterium tuberculosis metabolism at acidic pH. Trends Microbiol 27:942–953. doi:10.1016/j.tim.2019.06.005 31324436 PMC6800632

[93] Sanchez KG, Prest RJ, Nicholson KR, Korotkov KV, Champion PA. 2022. Functional analysis of EspM, an ESX-1-associated transcription factor in Mycobacterium marinum. J Bacteriol 204:e00233-22. doi:10.1128/jb.00233-22 36448785 PMC9765225

[94] Reynolds TS, Hu DD, Weaver SD, Ronck EC, Mishra SJ, Champion MM, Blagg BSJ. 2025. Proteomic analysis of Hsp90β-selective inhibitors against triple-negative breast cancer to gain a mechanistic insight. Mol Cell Proteomics 24:101043. doi:10.1016/j.mcpro.2025.101043 40714319 PMC12446539

[95] Buehl CJ, Deng X, Liu M, McAndrew MJ, Hovde S, Xu X, Kuo M-H. 2014. Resolving acetylated and phosphorylated proteins by neutral urea Triton-polyacrylamide gel electrophoresis: NUT-PAGE. BioTechniques 57:72–80. doi:10.2144/000114197 25109292 PMC4142444

