# Supplementary material for: pH-responsive substrate switching in mycobacterial type VII ESX secretion
Source: mSphere. 2026 Apr 29;11(5):e00056-26. doi: 10.1128/msphere.00056-26 (PMC13203967; doi:10.1128/msphere.00056-26)
Supplement: Supplemental Material — Supplemental figures, methods, and tables. [file msphere.00056-26-s0002.pdf]

Supplementary Materials for

# pH-responsive substrate switching in mycobacterial Type VII ESX secretion

Owen A. Collars[1], Richard E. Hernandez[1,2],Simon D. Weaver[1,2], Rebecca J. Prest[1]†, Caleb Manu[1], Gopinath Viswanathan[3], Rachel M. Cronin[1]‡, Bradley S. Jones[1], David J. Tobin[3], Matthew M. Champion[2], Patricia A. Champion[1]*

Corresponding author: pchampio@nd.edu

**The PDF file includes:**

Materials and Methods

Figs. S1 to S7
**Fig. S1.** *M. marinum* strains lacking Group 1-III ESX-1 substrates do not undergo bacteriolysis in the macrophage cytoplasm.
**Fig. S2.** Genotyping PCR to confirm *M. marinum* double deletion strains.
**Fig. S3.** Red blood cell lysis assay demonstrating complementation of strains that were non-hemolytic at pH 5.0.
**Fig. S4.** Total PDIM and PGL levels are not impacted by pH or detergent.
**Fig. S5.** EsxB is secreted from *M. marinum* following growth in 7H9 media.
**Fig. S6.** Whisker plots of levels of ESX-1-components in cell associated fractions.
**Fig. S7.** Volcano plots of ESX-1-dependent secretion in 7H9 media.

Tables S1 to S2
**Table S1.** Bacterial strains used in this study
**Table S2.** Oligonucleotides used in this study

Dataset S1

References

**Materials and Methods**

Growth and Generation of Bacterial Strains

All *M. marinum strains* were derived from the M strain (ATCC BAA-535). *M. marinum* strains were maintained in Middlebrook 7H9 defined broth (Sigma-Aldrich, St Louis MO) supplemented with 0.5% glycerol and 0.1% Tween-80 (Fisher Scientific, Pittsburgh PA) or on Middlebrook 7H11 agar (Sigma-Aldrich) plates supplemented with 0.5% glycerol and 0.5% glucose at 30°C. To buffer the pH of the liquid growth media, 7H9 defined broth was supplemented with 100mM 3-(*N*-morpholino)propanesulfonic acid (MOPS) (ThermoFisher, Waltham, MA) and buffered to pH 6.8 or pH 5. Tyloxapol (Chem-Impex, Wood Dale IL) was supplemented as indicated in the figures and figure legends. Agar plates and broth were supplemented with 20 µg/mL kanamycin (IBI Scientific, Peosta IA), 50 µg/mL hygromycin (EMD Millipore, Billerica MA), or 60µg/mL X-gal (Millipore) as necessary. *E. coli* DH5α strains were grown at 37°C in LB (Luria-Bertani) media (VWR, Radnor PA). As necessary, antibiotics were added to agar plates or broth at the following concentrations: 50 µg/mL kanamycin, 200 µg/mL hygromycin, or 200 µg/mL ampicillin (ThermoFisher).

Nomenclature

Nomenclature in this work follows the convention for ESX genes set by Bitter et al. (1)

Generation of Bacterial Strains and Plasmids

Deletion strains of *M. marinum* were constructed using allelic exchange using the p2NIL vector (Addgene plasmid number 20188; a gift from Tanya Parish) and the pGOAL19 vector (Addgene plasmid number 20190; a gift from Tanya Parish) exactly as previously described (2-5). The p2NIL/GOAL vectors used to generate deletions of single ESX-1 substrate genes were previously published (2, 6, 7) were used to generate the double deletion strains. Complementation plasmids expressing ESX-1 genes behind the mycobacterial optimal promoter were previously published (2). All double deletion strains were verified using PCR. After approximately 5 days of growth, 500µl of *M. marinum* culture were collected and lysed using a mini bead beater (Biospec Products, Batesville, OH) followed by centrifugation to remove cell lysis products. 1µl of lysate was used in 10µl PCR reactions to confirm the genotype of each strain. The resulting PCR products were separated on TAE agarose gels stained with ethidium bromide (VWR) and imaged using a Gel Doc EZ imager (Bio-Rad, Hercules, CA) and Image Lab software (Bio-Rad). The PCR verification is shown in Fig. S4. PCR products were sequenced to verify the deletion junctions using targeted DNA sequencing at the Genomics and Bioinformatics Core Facility at the University of Notre Dame.

Hemolysis Assay

*M. marinum* strains were grown in Middlebrook 7H9 broth and 0.1% Tween-80 to mid-log phase. 24 hours prior to the hemolysis assay, the bacteria were sub-cultured into 7H9 broth buffered with 100mM MOPS to pH 6.8 or pH 5.0 overnight. Where applicable, varying concentrations of tyloxapol (from 0.000625% through 0.01355%, or 0.2%, as indicated in the figures and text), or 200µM oleic acid (ThermoFisher, Waltham MA) was added to cultures at the time of subculturing. The number of bacterial cells in each sample were normalized to the optical density ($OD_{600}$). *M. marinum* cells were washed three times with 500µl PBS and resuspended in 300µl PBS. Sheep red blood cells (sRBCs, Hardy Diagnostics, Santa Maria CA) were diluted 1:10 in PBS, and washed to remove lysed cells. sRBCs were resuspended in 500µl PBS. Washed bacteria were mixed with 100µl of sRBCs, collected by centrifugation to initiate interaction, and incubated at 30 °C for 1.5 hours. The optical density of 405nm ($OD_{405}$) of each sample was read in technical triplicate in a 96-well plate on a SpectraMax ABS plate reader (Molecular Devices, San Jose, CA). For all hemolysis assays, incubation of sRBCs with water was a maximal lysis positive control. Incubation of sRBCs with PBS alone, or with the $\Delta$*eccCb*$_1$ *M. marinum* strain, serve as cell-free and bacterial negative controls.

RNA Extraction

*M. marinum* was grown in 5 ml of Middlebrook 7H9 media (Sigma Aldrich) + 0.1% Tween-80 (Fisher Scientific, Pittsburgh PA) for three days, then moved to 25 ml Middlebrook 7H9 + 0.1% Tween-80 for two days. *M. marinum* strains were diluted to $OD_{600}$ of 0.8 in Sauton's defined broth + 0.01% Tween-80 and grown for 24 hours at 30°C. After 24 hours, the *M. marinum* cells were collected using centrifugation and resuspended in Sauton's buffered to pH 6.8 or pH 5.0 with 100mM MOPs + 0.01% Tween-80 for 24 hours. Bacterial cells were collected by centrifugation of 15 ml of culture. The media was removed and the resulting

cell pellets were frozen. Thawed *M. marinum* cell pellets were resuspended in Qiagen RLT buffer (Qiagen, Hilden, Germany) and supplemented with 1% β-mercaptoethanol (Sigma-Aldrich). Whole cell *M. marinum* lysates were generated by bead beating the resuspended cells using a Biospec Mini-BeadBeater-16. Total RNA was extracted from clarified lysates using the RNeasy Mini Kit (Qiagen), according to manufacturer's instructions.

RT-qPCR

RT-qPCR was performed exactly as in Nicholson et al. Briefly, 500 ng of RNA was treated with Promega RQ1 DNase (Promega, Madison WI) according to manufacturer instructions and supplemented with 5mM $MgCl_2$ and 10mM $CaCl_2$. 1μl of DNase treated RNA was converted to cDNA using random hexamers (IDT, Coralville IA) and Superscript II (SSII) Reverse Transcriptase (Invitrogen, Carlsbad CA) according to manufacturer's instructions. cDNA was quantified using a NanoDrop 2000 (ThermoFisher). qRT-PCR reactions were prepared using 250ng of cDNA mixed with SYBR Select Master Mix (Applied Biosystems, Carlsbad, CA) and 1μM of each oligonucleotide as listed in Table S2. All transcripts were normalized to *sigA*. All RT-qPCR reactions were run using Applied Biosystems MicroAmp Fast 96 well plates (0.1mL, Life Technologies) in the QuantStudio 3 RealTime PCR System (ThermoFisher) using the cycling conditions in (8). The resulting data were analyzed using ΔΔCt comparisons and were normalized to WT transcript abundance exactly as in (8).

Bacteriolysis

Immortalized IFNAR$^{-/-}$ (iIFNAR$^{-/-}$) bone marrow derived macrophages (gift of Dr. J.D. Sauer, (9)) were maintained in immortalized Macrophage Media (iMM), consisting of RPMI 1640 (Gibco, Frederick MD) supplemented with 10% heat-inactivated fetal bovine serum (FBS, Avantor Seradigm, Radnor PA), 1% sodium pyruvate (Gibco), and 0.1% β-mercaptoethanol (Gibco). Cells were maintained at 37 °C with 5% $CO_2$. iIFNAR-/- macrophages were seeded at 5 × $10^5$ cells/mL in tissue culture-treated 24-well plates (Greiner Bio-One, Kremsmünster, Austria) for 24 h. The macrophages were infected at a multiplicity of infection (MOI) of 20 with single-cell suspension of each *M. marinum* deletion and complementation strain carrying pTAS1 plasmid (5). Infections proceeded for 2 hours at 37°C with 5% $CO_2$, after which 100 μg/mL gentamicin (Research Products International, Mount Prospect IL) was added for 4 h at 37 °C with 5% $CO_2$ to kill extracellular *M. marinum*. Monolayers were washed with PBS, and fresh iMM was added to each well. At 24 hpi, the media was removed, and 300 μL of TNT lysis buffer (20 mM Tris base, 100 mM NaCl, 1% Triton X-100 [pH 8]) was added to each well and incubated for 10 min at room temperature to lyse the cells. Lysates were transferred in triplicate from each well to a white-bottom 96-well plate (Greiner Bio-One). Firefly luciferase substrate (0.02M Tricine, 500mM $MgSO_4 \cdot 7H_2O$, 500mM EDTA, 1M DTT, 100mM ATP, 25mg/mL acetyl-CoA lithium salt, 0.14mg/mL luciferin, 2M NaOH, 50mM magnesium carbonate hydroxide) was added to each well, and luciferase activity was measured using a Synergy H1 multimode microplate reader (BioTek, Winooski VT).

ESX-1 Secretion Assay

*M. marinum* strains were grown in 7H9 media supplemented with glucose, glycerol and 0.1% Tween-80 until turbid, for ~5 days. The cultures were diluted to an $OD_{600}$ of 0.8 in 50 mls of 7H9 broth with 0.5% glycerol, but without glucose. When necessary, the media was buffered to pH 6.8 or pH 5 with 100mM MOPs, supplemented with 0.01% Tween-80. The cells were grown for 48 hours at 30°C. *M. marinum* cells were collected using centrifugation. The resulting supernatant was filtered through 0.2μm Nalgene Stericups with polyethersulfone (PES) filters and concentrated by ultrafiltration in a 3,000- molecular-weight-cutoff Amicon filter (Millipore). Cell-associated proteins were extracted from cells by lysis in PBS with a Biospec Mini-BeadBeater-16. Protein concentrations for the resulting fractions were determined using the Pierce MicroBCA kit (Thermo Scientific). Desired protein quantities were precipitated in four times volume of acetone (Thermo-Fisher) for one hour. Samples were then centrifuged at 14K rpm for 10 minutes at 4 degrees Celsius. Acetone was then removed and pelleted protein samples were air dried and resuspended in 16μL of PBS prior to SDS-PAGE and Immunoblotting.

Immunoblotting Analysis

Unless otherwise noted, 20μg of protein were loaded onto 4-20% TGX Gradient Gels (Bio-Rad) and separated by gel electrophoresis before transferring to Amersham Protran 0.2 μm nitrocellulose (Cytiva, Marlborough MA).Western blots were imaged using a Licor C-Digit digital developer (LICORbio,

Lincoln NE) and analyzed using Image Studio 6.1 (LICORbio). All antibodies were diluted in 5% nonfat dry milk in PBS with 0.1% Tween 20. RNAP polymerase subunit β (RNAP-β, ab12087; Abcam, Cambridge UK) was diluted 1:20,000. The following reagents were obtained through BEI Resources, NIAID, NIH: Polyclonal anti-*Mycobacterium tuberculosis* Mpt32 (gene *Rv1860*, antiserum, rabbit) NR-13807, was used at 1:20,000. The EspE antibody (1:5,000 dilution, rabbit, epitope: CGQQATLVSDKKEDD) was generated by GenScript (Piscataway NJ) based on this publication (10). The EspA antibody (1:1,000 dilution, rabbit, epitope: AG-SKGATSKKYSEG ) was generated by GenScript based on this publication (11). Horseradish peroxidase (HRP)-conjugated goat anti-mouse immunoglobulin secondary antibody (Bio-Rad) was used at 1:5,000 for detection of the RNAPβ antibody. HRP-conjugated goat anti-rabbit IgG secondary antibody (Bio-Rad) was used at a dilution of 1:5,000 for detection of the Mpt-32, EspE, and EspA antibodies.

Proteomics/LC-MS

LC-MS pure reagents (water, ethanol, acetonitrile, and methanol) were purchased from J. T. Baker (Radnor, PA). Iodoacetamide (IAA) was purchased from MP Biomedicals (Solon. OH). All other reagents were obtained from Millipore-Sigma (St. Louis, MO), unless specified. S-Trap Micro devices were obtained from Protifi (Huntington, NY). Trypsin Gold was purchased from Promega (Madison, WI). Hydrophilic–lipophilic balance (HLB) solid-phase extraction (SPE) cartridges (1 cc/10 mg) from Waters (Milford, MA) were used to desalt peptide samples prior to analysis on a timsTOF Pro 2 from Bruker Scientific LLC (Billerica, MA).

Cell-associated and secreted cell lysate samples were prepared for LC-MS analysis, as described (13, 14). Twenty-five micrograms of each sample was prepared in 140 mM triethylammonium bicarbonate (TEAB), 10% sodium dodecyl sulfate (SDS), and 100 mM tris(2-carboxyethyl)phosphine (TCEP). Samples were heated for 10 minutes at 95°C, cooled, and then IAA was added to 100 mM IAA for 30 minutes in the dark.

Samples were acidified with *o*-phosphoric acid to a final concentration of 1.1% in 55 µL and then flocculated with 350 µL binding buffer containing 90% methanol and 10% 1M TEAB (v/v). Samples were passed through S-Trap Micro filters and followed by two washes with 80 µL of binding buffer and once with 80 µL of 1:1 methanol/chloroform solution (v/v) in between. A new collection tube was added, and 1 µg of trypsin in 100 µL 100 mM TEAB was added. Samples were wrapped in parafilm to prevent evaporation and incubated at 37°C overnight. Digested peptides were eluted by centrifugation through the filter, followed by two 80 µL elutions with 0.1% formic acid in water and one 80 µL elution with 0.1% formic acid in 50% acetonitrile. Eluted peptides were vacuum-concentrated for 20 minutes (to remove acetonitrile) and then desalted using 1 cc/10 mg HLB SPE cartridges following the manufacturer's specifications and then dried by a vacuum concentrator prior to analysis.

Desalted peptides were resuspended in 0.1% formic acid and water to a concentration of 1 mg/mL. 500 ng of each secreted fraction and 250 ng of each cell-associated fraction was injected in duplicate onto a nanoElute 2 and timsTOF Pro 2 LC-MS system. Each sample was prepared in biological quadruplicate with technical duplicates. Sixty-minute gradient methods were used on a 75 µm x 150 mm PepSep column with the C18 ReproSil AQ stationary phase at 1.9 µm particle size, 120 Å pore. Samples were loaded in one or two microliter injections of 250 ng/µL. Nano-ESI was used with a spray voltage of 1,700 V. MS was set to the parallel accumulation, serial fragmentation data-independent mode (PASEF-DIA) with a mass range of 401–1,225 m/z, ion mobility range of 0.73–1.40 v*s/cm$^2$, and ramp and accumulation times of 100 mS. For the secreted fraction injections, each cycle consisted of 19 MS/MS ramps with 26 Da mass windows resulting in a 2.12s cycle time. For the pellet fractions, each cycle consisted of 21 MS/MS ramps for a cycle time of 2.33s. MS/MS collision energy settings were set to ramp from 20 eV at 0.7 ion mobility to 70 eV at 1.5 ion mobility. Instrument tune parameters were set to default for proteomic studies with the following differences: quadrupole low mass set to 20 m/z and focus pre-TOF pre-pulse storage set to 5 µs. Raw data files and instrument parameters were uploaded to massIVE and Proteome Xchange, details in data availability section below.

Data Analysis

Bruker data files (.d) were searched using Spectronaut (v19.2.240905.6263) with directDIA Analysis using the *Mycobacterium marinum (strain ATCC BAA-535 / M)* reference proteome (UP000001190_216594; 5418 entries) downloaded on 2025-11-24. BGS factory settings were used. The cleavage rules were set to Trypsin/P with two allowed missed cleavages. Carbamidomethyl (C) was set as a fixed modification and Acetyl (Protein N-term), Deamidation (NQ), Gln->pyro-Glu, Glu->pyro-Glu, and

Oxidation (M) were set as variable modifications. The pellet samples and supernatant samples were searched and analyzed separately. Protein results were exported as .tsv files, with the PG.ProteinAccession field (uniprot ID) used as the unique protein identifier, and the PG.Log2Quantity field used as the protein quantity ($Log_2$ transformed). Full search parameters were uploaded to massIVE and Proteome Xchange, details in data availability section below.

The following analysis was performed in R (v4.3.1). For each protein, the two technical replicates for each condition were averaged to produce a single quantitative value ($Log_2$ transformed) for each biological replicate. The remaining analysis was performed using these values (n=4 per strain/pH combination) so that statistical significance is based on variance between biological replicates. Each biological replicate was normalized with the center.median function from QFeatures (v1.10.0). Differential expression analysis was performed with the limma (v3.56.2) empirical Bayesian method (15), defining each strain/pH combination as a single coefficient with 4 replicates and using a contrast matrix to define the pairwise comparisons between conditions (e.g. "WT_5 – WT_6" to compare the WT at pH 5.0 condition to the WT at pH 6.8 condition). Multiple hypothesis testing correction was performed with the Benjamini-Hochberg (B-H) method built into the limma package. Volcano plots were created by plotting $-Log_{10}$ of this corrected *p-value* against the $Log_2$ fold change (LFC) with visual cutoffs set at LFC of –1 and 1 and a significance cutoff at <0.05 B-H adjusted *p-value.* Specific proteins of interest were pulled out of this dataset (e.g. ESX substrates) and plotted as box and whisker plots showing each biological replicate as one point, but annotated with the statistical significance from B-H adjusted p-values as described above (ns = not significant, * = adj.p.value < 0.05, ** = adj.p.value < 0.01, *** = adj.p.value < 0.001). Differential abundance outputs are included in the Dataset.

Data availability

Raw data files, search parameters, and instrument method parameters were deposited in massIVE (identifier MSV000100192) and Proteome Xchange (identifier PXD071871).

Lipid Extraction and TLC Analysis.

*M. marinum* strains were grown in 5 mL of 7H9 supplemented with 0.2% Tween-80. The mycobacterial cultures were scaled up to a 50 mL with a starting OD600 value to 0.8 in 7H9 media buffered to pH 6.8 or pH 5.0, with either 0.1% percent Tween 80 (Fisher Scientific) or 0.2% tyloxapol (Chem-Impex International) and grown for two days at 30°C with shaking. Bacterial cells were collected after 48 hours via centrifugation, and total lipids were extracted and TLC analysis were performed as in (16) with the following changes. For PDIM analysis, spotted total lipids were migrated in petroleum ether (Sigma-Aldrich, St. Louis, MO) and ethyl acetate (Sigma-Aldrich) at a 50:1 ratio twice, instead of three times.

Expression profiling of *M. marinum* genes in zebrafish granulomas

RNA-expression analysis of *M. marinum* genes within adult zebrafish granulomas relative to in vitro broth cultures was carried out with the RNA-seq data obtained from (17). The data were from 4 biological replicates for adult fish infection and 3 biological replicates for in vitro growth. For *in vitro* conditions, total RNA was isolated from *M. marinum* grown to an $OD_{600}$ of ~ 1.0 in 7H9 complete media supplemented with 10% OADC (50 g/L BSA, 0.5% oleic acid, 20 g/L dextrose, 8.5 g/L NaCl) and 0.05% Tween-80. Cultures were incubated at 33°C with shaking at 150 RPM. RNA extraction was performed in triplicate. For each biological replicate, around 400 granulomas were dissected from 8-12 wild-type adult zebrafish infected with *M. marinum* at 14 dpi. Total RNA from these granulomas was enriched for *M. marinum* mRNA by following a two-step approach. First, zebrafish mRNA was removed from total RNA using two oligo dT captures (Kapa mRNA HyperPrep Kit #KK8581). They were subsequently treated with additional custom rRNA depletion probes for zebrafish and *M. marinum*, reverse transcribed into cDNA, and processed into sequencing libraries. Total RNA from in vitro *M. marinum* log phase cultures were treated similarly. The libraries were sequenced on the Illumina NovaSeq 6000 S Prime flow cell to generate 150 bp paired-end reads. The reads were pseudoaligned to the *M. marinum* transcriptome index built from the corresponding transcriptome (GCA_000018345.1 (ASM1834v1)) using Kallisto (v. 0.46.1), to quantify transcript abundances. Differential gene expression analysis was performed using the DESeq2 package.

*Galleria mellonella* Infections

*Galleria mellonella* (Greater Wax Moth larvae, “wax worms” from waxworms.net ) infections with *M. marinum* were performed exactly as previously published (16). Briefly, *M. marinum* single cell suspensions were generated at a concentration of $1x10^7$ / 5 µL. Larvae were infected with 5µl of *M. marinum* suspension in triplicate in groups of 10 larvae, resulting in 30 larvae used per strain per biological replicate. After injection, larvae were incubated at 30°C for the infection over 8 days. Death was recorded daily.

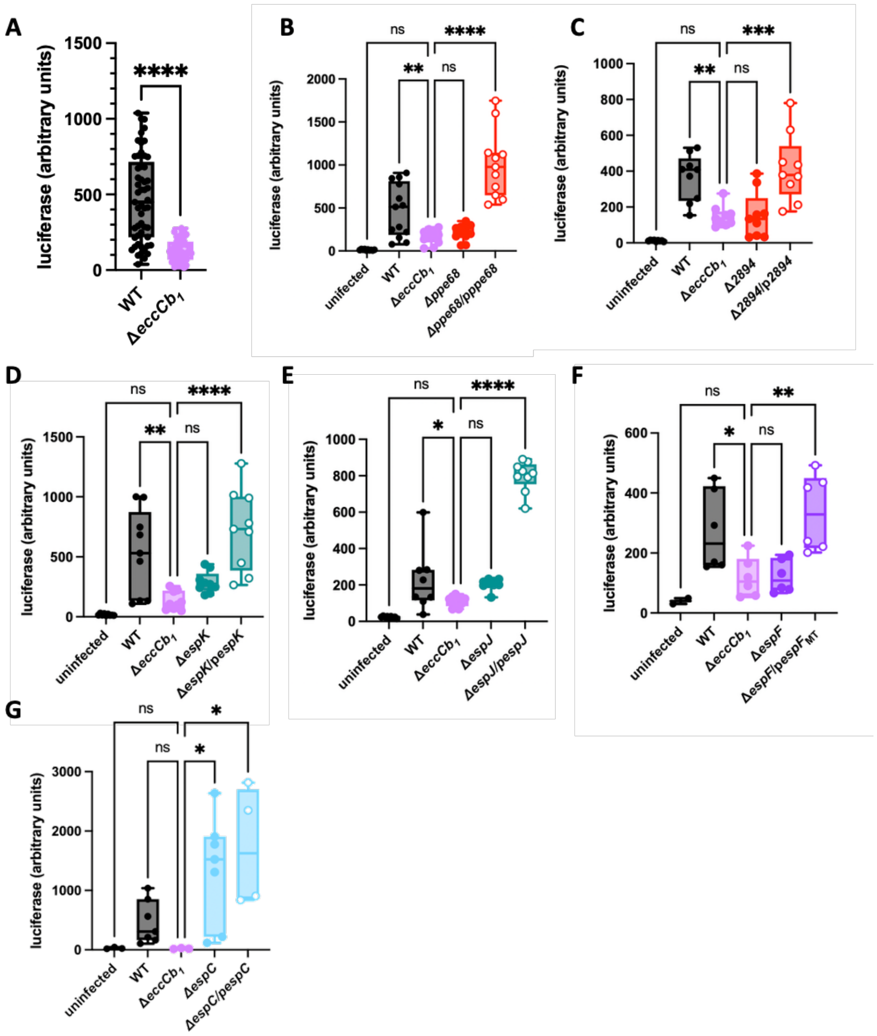


**Fig. S1.** ***M. marinum*** **strains lacking Group 1-III ESX-1 substrates do not undergo bacteriolysis in the macrophage cytoplasm. (A)** WT/ pTAS1 and $\Delta eccCb_1$/ pTAS1 reporter strains across all infections represented in this figure. Significance was determined using a t-test with a Welch's correction. **** *P*<.0001. The requirement of each ESX-1 substrate gene on bacterial lysis in the macrophage cytoplasm. The WT/pTAS1 strain is a positive control for bacteriolysis. The $\Delta eccCb_1$/pTAS1 strain is a negative control, and is retained in the phagosome because it lacks a functional ESX-1 secretion system. Bacteriolysis of *M. marinum* lacking (B) the *ppe*68 Group I gene,(C) the *MMAR_2894* Group I gene, (D) the *espK* Group II gene, (E) the *espJ* Group II gene, (F) the *espF* Group III gene (G) and the *espC* Group IV gene. For (B)-(G) significance was determined using an ordinary one-way ANOVA followed by a Dunnett's multiple comparison test against the phagosomal $\Delta eccCb_1$ strain (B) ANOVA: *P<.0001,* Dunnett's: **** *P*<.0001, ** *P*=.0089, (C) ANOVA: *P<.0001,* Dunnett's: *** *P*=.0001, ** *P*= .0018, (D) ANOVA: *P<.0001,* Dunnett's: **** *P*<.0001, ** *P*=.0020, (E) ANOVA: *P<.0001,* Dunnett's: **** *P*<.0001, * *P*=.0273, (F) ANOVA: *P=.0011,* Dunnett's: ** *P*=.0042, * *P*=.0461, (G) ANOVA: *P=.0046,* Dunnett's: * *P*=.0321 (vs *Δ*espC), * *P*=.0126 (vs Δ*espC/pespC).* Each data point is a biological replicate, representing the mean of three technical replicates. Open circles are complementation strains.

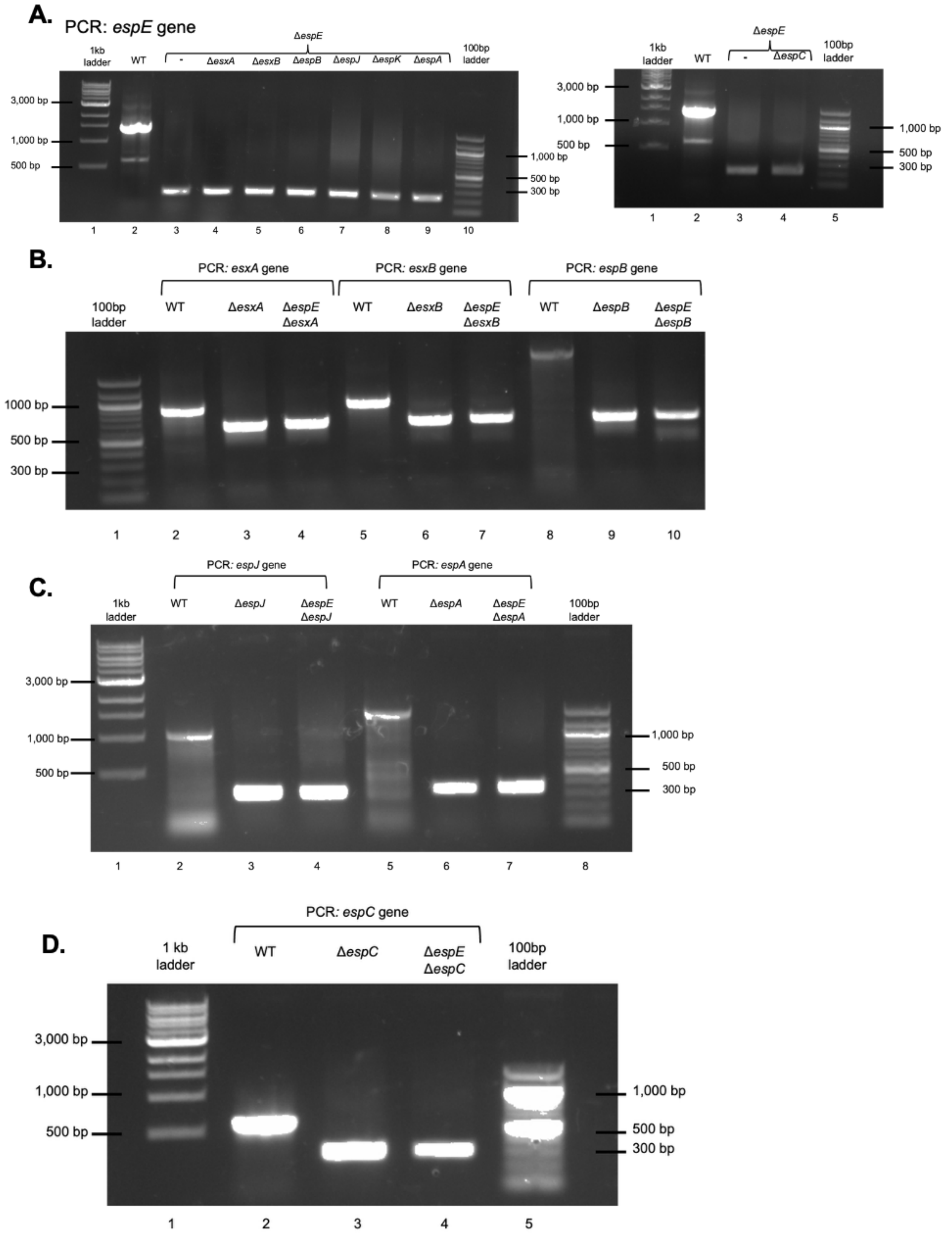
A.
PCR: espE gene
ΔespE
1kb ladder
WT
-
ΔesxA
ΔesxB
ΔespB
ΔespJ
ΔespK
ΔespA
100bp ladder
3,000 bp
1,000 bp
500 bp
1,000 bp
500 bp
300 bp
1 2 3 4 5 6 7 8 9 10
ΔespE
1kb ladder
WT
-
ΔespC
100bp ladder
3,000 bp
1,000 bp
500 bp
1,000 bp
500 bp
300 bp
1 2 3 4 5
B.
PCR: esxA gene
PCR: esxB gene
PCR: espB gene
100bp ladder
WT
ΔesxA
ΔespE ΔesxA
WT
ΔesxB
ΔespE ΔesxB
WT
ΔespB
ΔespE ΔespB
1000 bp
500 bp
300 bp
1 2 3 4 5 6 7 8 9 10
C.
PCR: espJ gene
PCR: espA gene
1kb ladder
WT
ΔespJ
ΔespE ΔespJ
WT
ΔespA
ΔespE ΔespA
100bp ladder
3,000 bp
1,000 bp
500 bp
1,000 bp
500 bp
300 bp
1 2 3 4 5 6 7 8
D.
PCR: espC gene
1 kb ladder
WT
ΔespC
ΔespE ΔespC
100bp ladder
3,000 bp
1,000 bp
500 bp
1,000 bp
500 bp
300 bp
1 2 3 4 5

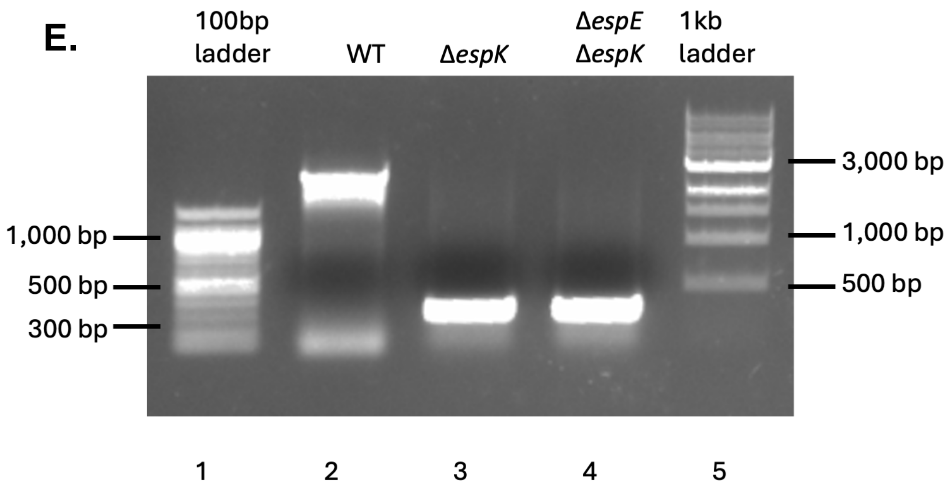


**Fig. S2. Genotyping PCR to confirm *M. marinum* double deletion strains.** Primers flanking the genomic locus of ESX-1 genes were used to check for the loss of individual genes in the Δ*espE* double deletion strains. (**A)** *espE* gene. Product: WT 1,462bp, Δ*espE* 205bp**. (B)** *esxA* gene (lane 2-4). Product: WT 890bp, Δ*esxA* 641bp. *esxB* gene (lane 5-7). Product: WT 890bp, Δ*esxB* 641bp. *espB* gene (lane 8-10). Product: WT 1933bp, Δ*espB* 601bp. (**C)** *espJ* gene (lane 2-4). Product: WT 1049, Δ*espJ* 239bp. *espA* gene (lane 5-7). Product: WT 1496, Δ*espA* 335bp. (**D)** *espC* gene. Product: WT 586bp, Δ*espC* 304bp. (**E)** *espK* gene. Product: WT 2574bp, Δ*espK* 294bp.

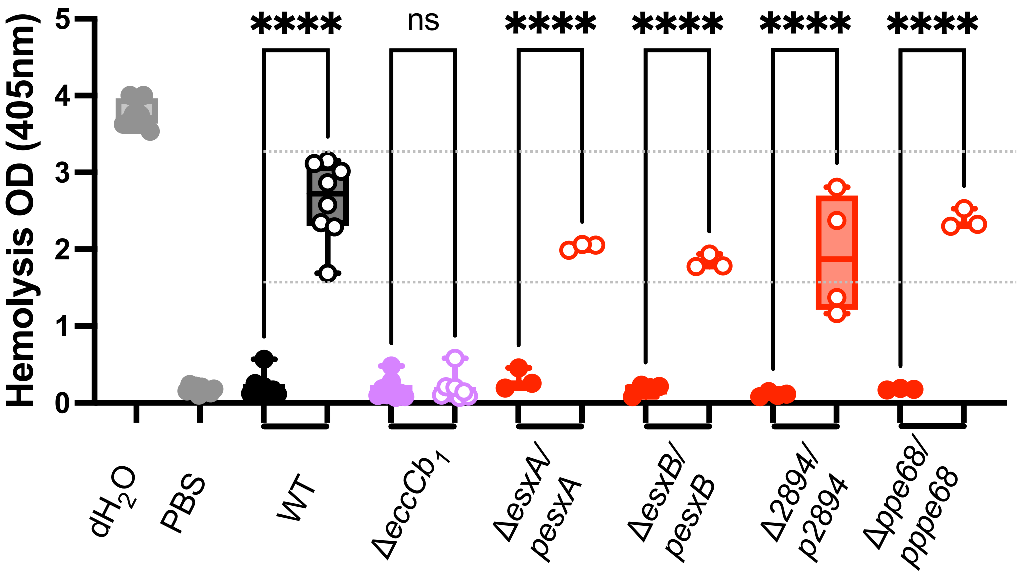


**Fig. S3. Complementation of hemolytic activity following growth at pH 5.0 in the presence of tylox-apol.** Hemolytic activity of *M. marinum* grown at 7H9 buffered to either pH 6.8 or pH 5.0 with 0.2% Tyloxapol for 24 hours. Each data point represents a biological replicate, which is the mean of three technical replicates. Significance was determined using a one-way ordinary ANOVA ($P$<0.0001) followed by a Tukey's multiple comparison test. Significance of strains grown at pH 5.0 relative to the same strain grown at pH 6.8 **** $P$<0.0001. Dotted lines indicate the range of WT- hemolytic activity.

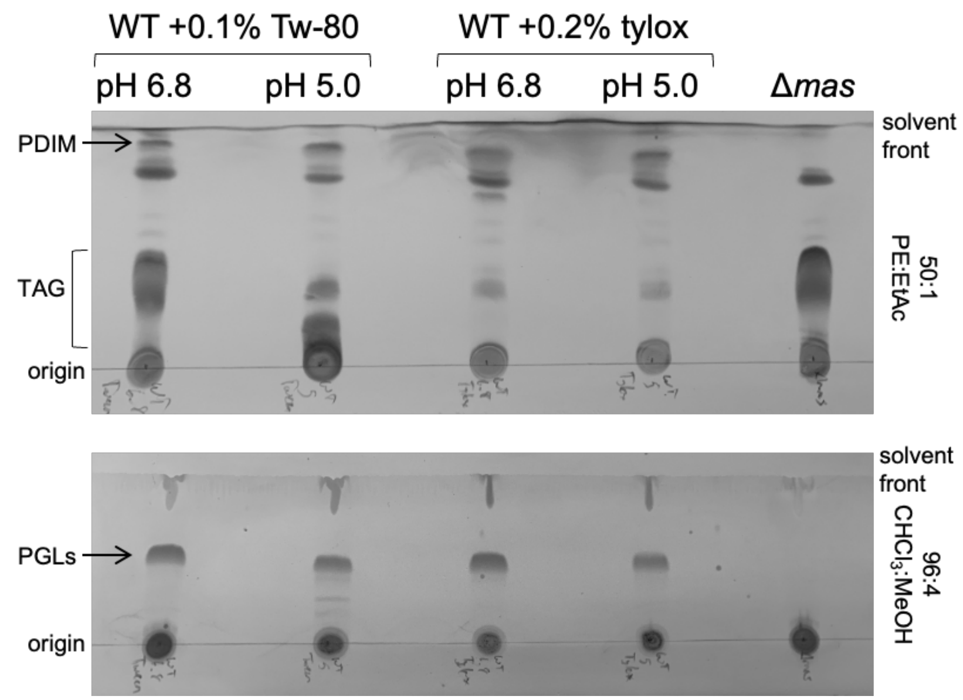


**Fig. S4. Total PDIM and PGL levels are not impacted by pH or detergent.** Thin-layer chromatography analysis of total mycobacterial lipids harvested from wild-type *M. marinum* M strain, cultured under the pH and detergent supplementation conditions indicated. The Δ*mas* strain does not produce PDIM or PGL and serves as a negative control for this assay. 6.0 µL of total lipids were migrated and analyzed on each silica plate. For PDIM lipids, petroleum ether and ethyl acetate were used as solvents at a 50:1 ratio. For PGL lipids, chloroform and methanol were used as solvents at a 96:4 ratio. The results shown are representative of three biological replicates.

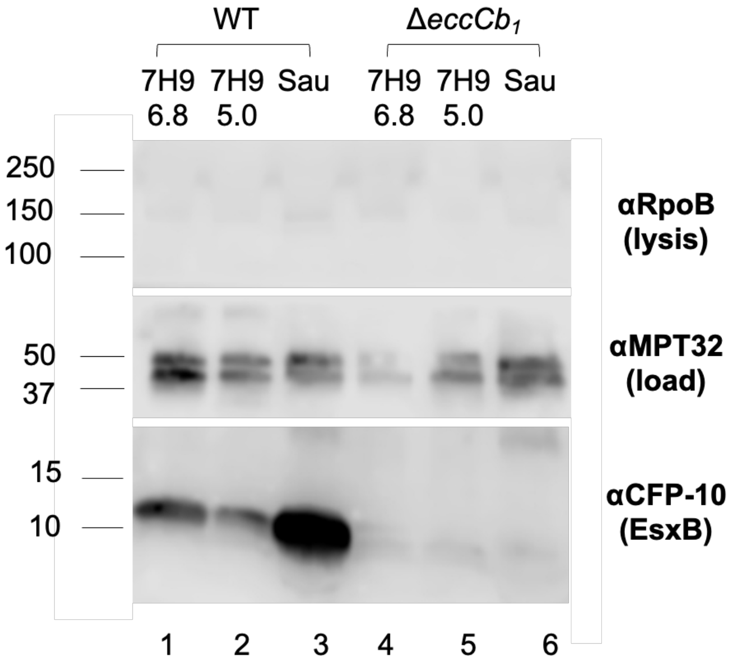


**Fig. S5. EsxB is secreted from *M. marinum* following growth in 7H9 media.** Western blot analysis of secreted protein fractions from the WT (lanes 1-3) or Δ*eccCb*$_1$ (lanes 4-6) strains following 48 hours of growth in either 7H9 media buffered to pH 6.8 or 5.0 vs Sauton's define media. 20 µg of protein was loaded in each lane, and resolved on a 4-20% mini protean TGX gel. RpoB is a measure of cell lysis. MPT-32 is protein secreted by the Sec secretion system and is a loading control for the secreted protein fraction. EsxB is a Group 1 substrate of the ESX-1 system. Data shown are representative of three independent experiments.

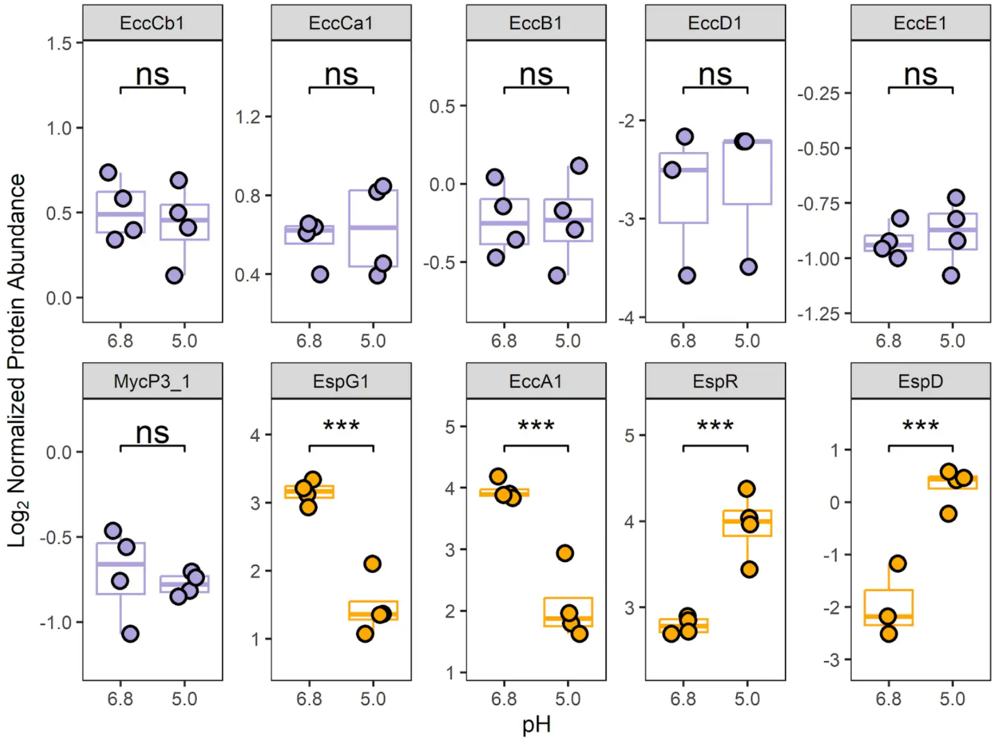


**Fig. S6. Whisker plots of levels of ESX-1-components in cell associated fractions.** Box and whisker plots show independent biological replicates (each is an average of two technical replicates) for each protein with the significance annotation from the Benjamini-Hochberg adjusted p-value (ns = not significant, * = adj.p.value < 0.05, ** = adj.p.value < 0.01, *** = adj.p.value < 0.001) as calculated using the biological replicates (n=4). Strain: WT, fraction: pellet. Light purple: core component. Orange: transcription factor or chaperone.

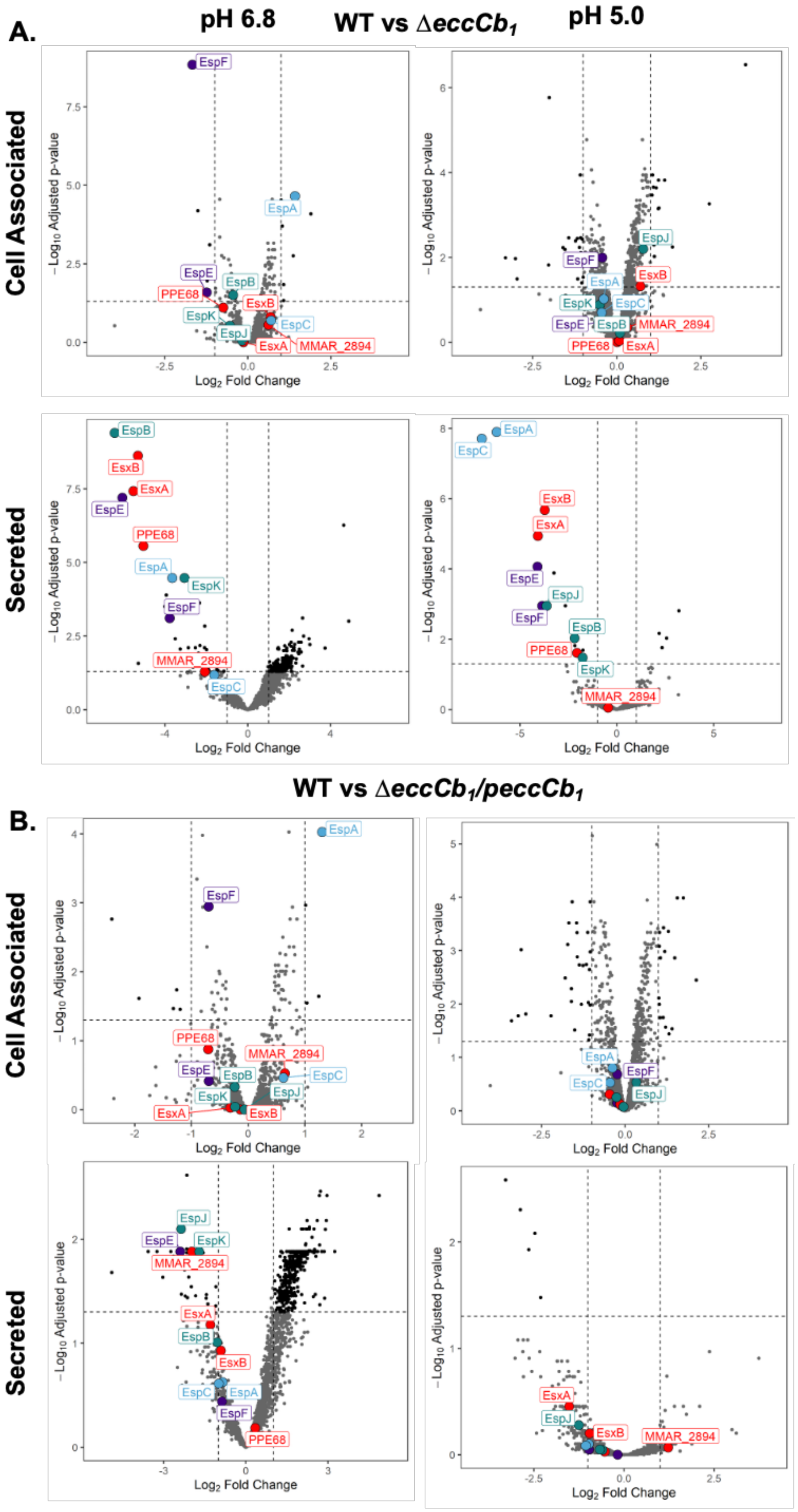
A.
pH 6.8
WT vs ΔeccCb1
pH 5.0
Cell Associated
Secreted
−Log10 Adjusted p-value
Log2 Fold Change
EspF
EspA
EspE
PPE68
EspB
EspK
EsxB
EspC
EspJ
EsxA
MMAR_2894
B.
WT vs ΔeccCb1/peccCb1

**C.**

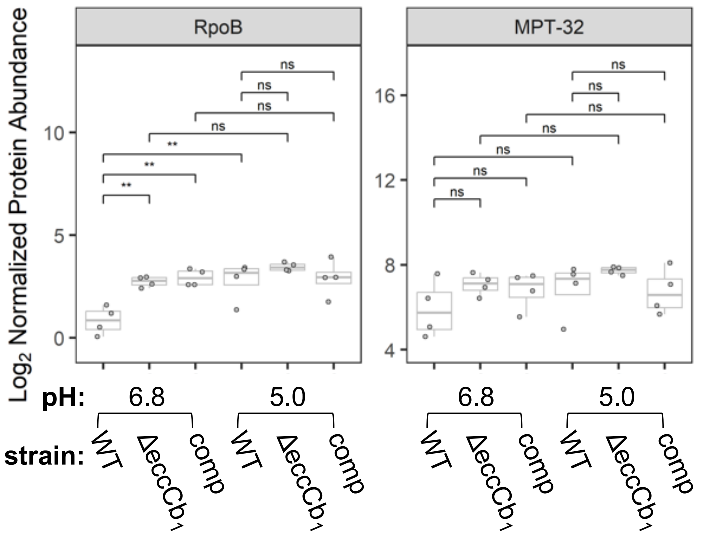


**Fig. S7. Volcano plots of ESX-1-dependent secretion in 7H9 media.** $Log_2$ Fold Change (LFC) of proteins from the WT strain vs (**A**) the Δ*eccCb₁* strain or the (**B**) Δ*eccCb₁*/p*eccCb₁* strain following growth at pH 6.8 and pH 5.0 plotted against the $-Log_{10}$ adjusted p-value for the cell associated or the secreted protein fractions. Dotted lines are drawn at LFC of -1 and 1, and a significance cutoff of adjusted p-value = 0.05. **(C)** Box and whisker plots show independent biological replicates (each is an average of two technical replicates) for each protein with the significance annotation from the Benjamini-Hochberg adjusted p-value (ns = not significant, ** = adj.p.value < 0.01 as calculated using the biological replicates (n=4).

Table S1. Bacterial strains used in this study

| ***M. marinum* strains** | | |
|---|---|---|
| **Name** | **Genotype** | **Reference** |
| *M. marinum* M strain | Wild type strain; parental strain | ATCC BAA-535 |
| Δ*eccCb*$_1$ | M with a deletion of the *eccCb*$_1$ gene | (3) |
| Δ*esxA* | M with a deletion of the *esxA* gene | (2) |
| Δ*esxB* | M with a deletion of the *esxB* gene | (2) |
| Δ*MMAR_2894* | M with a deletion of the *MMAR_2894* gene | (7) |
| Δ*ppE68* | M with a deletion of the *ppE68* gene | (2) |
| Δ*espB* | M with a deletion of the *espB* gene | (18) |
| Δ*espJ* | M with a deletion of the *espJ* gene | (2) |
| Δ*espK* | M with a deletion of the *espK* gene | (2) |
| Δ*espE* | M with a deletion of the *espE* gene | (6) |
| Δ*espF* | M with a deletion of the *espF* gene | (6) |
| Δ*espA* | M with a deletion of the *espA* gene | (2) |
| Δ*espC* | M with a deletion of the *espC* gene | (2) |
| Δ*espA/espA* | Δ*espA* with the p*espA* plasmid integrated at the *attB* site | (2) |
| Δ*espC/pespACD* | Δ*espC* with the p*espACD* plasmid integrated at the *attB* site | (2) |
| Δ*esxA/pesxBA* | Δ*esxA* with the p*esxBA* plasmid integrated at the *attB* site | (2) |
| Δ*esxB/pesxBA* | Δ*esxB* with the p*esxBA* plasmid integrated at the *attB* site | (2) |
| Δ*2894/p2894* | Δ*2894* with the p*2894* plasmid integrated at the *attB* site | (2) |
| Δ*ppe68/pppe68* | Δ*ppe68* with the p*ppe68* plasmid integrated at the *attB* site | (2) |
| Δ*espE*Δ*esxA* | M strain with a deletion of *espE* and *esxA* | This study |
| Δ*espE*Δ*esxB* | M strain with a deletion of *espE* and *esxB* | This study |
| Δ*espE*Δ*espB* | M strain with a deletion of *espE* and *espB* | This study |
| Δ*espE*Δ*espJ* | M strain with a deletion of *espE* and *espJ* | This study |
| Δ*espE*Δ*espK* | M strain with a deletion of *espE* and *espK* | This study |
| Δ*espE*Δ*espA* | M strain with a deletion of *espE* and *espA* | This study |
| Δ*espE*Δ*espC* | M strain with a deletion of *espE* and *espC* | This study |
| Δ*espE*Δ*espF* | M strain with a deletion of *espE* and *espF* | This study |
| Δ*espA*Δ*espF* | M strain with a deletion of *espA* and *espF* | This study |
| Δ*espC*Δ*espF* | M strain with a deletion of *espC* and *espF* | This study |
| Δ*espC*Δ*espA* | M strain with a deletion of *espC* and *espA* | This study |
| WT/pTas1 | M strain bearing the episomal pTas1 reporter plasmid, which includes the luc+ gene expressed behind CMV enhancer, immediate early promoter, KanR | (5) |
| Δ*eccCb*$_1$/pTas1 | Δ*eccCb*$_1$ strain bearing the pTas1 plasmid | (5) |
| Δ*ppe68*/pTas1 | Δ*ppE68* strain bearing the pTas1 plasmid | This study |
| Δ*ppe68*/*pppe68,* pTas1 | Δ*ppE68/pppe68* strain bearing the pTas1 plasmid | This study |
| Δ*2894*/pTas1 | Δ*2894* strain bearing the pTas1 plasmid | This study |
| Δ*2894*/p*2894,* pTas1 | Δ*2894/p2894* strain bearing the pTas1 plasmid | This study |
| Δ*espK*/pTas1 | Δ*espK* strain bearing the pTas1 plasmid | This study |
| Δ*espK*/*pespK,* pTas1 | Δ*espK/pespK* strain bearing the pTas1 plasmid | This study |
| Δ*espJ*/pTas1 | Δ*espJ* strain bearing the pTas1 plasmid | This study |
| Δ*espJ*/*pespJ,* pTas1 | Δ*espJ/pespJ* strain bearing the pTas1 plasmid | This study |
| Δ*espF*/pTas1 | Δ*espJ* strain bearing the pTas1 plasmid | This study |
| Δ*espF*/pespF$_{MT}$, pTas1 | Δ*espJ/pespJ* strain bearing the pTas1 plasmid | This study |
| Δ*espC*/pTas1 | Δ*espC* strain bearing the pTas1 plasmid | This study |
| Δ*espC*/*pespC,* pTas1 | Δ*espC/pespC* strain bearing the pTas1 plasmid | This study |
| Δ*mas* | M strain with a deletion of *mas* | (16) |
| | | |

**Table S2. Oligonucleotides used in this study**

| Name | Sequence (5'->3') | Application/Reference |
|---|---|---|
| ORP 67 | CGGACCTTGGTGCTGTGCG | Confirm *espE* deletion, (19) |
| ORP 68 | GGGTTTGGCTCGCTATGGC | |
| OMF 170 | TCGTCAACACGAACAGACTTCCC | Confirm *esxA and esxB* deletion, (18) |
| OMF 171 | GTCATCTGGAGGTCCGGAACC | |
| OMF 214 | TAATCGGCGAGACCGTGAACC | Confirm *espB* deletion,(2) |
| OMF 215 | GTCCAAGGCCGACTGGAACC | |
| OMF 439 | AAACATCCGATTCTGAGTCACCGGC | Confirm *espJ* deletion, (2) |
| OMF 440 | GTTGGATGTCGCCGGTAACACC | |
| ORC 34 | TTGATCACCAAGCCGAGGAC | Confirm *espA* deletion, (2) |
| ORC 35 | TTAGCCCAGAGGTGACTTCG | |
| OLC 9 | GCGACCAGCAAGAAGTACTC | Confirm *espC* deletion, (2) |
| OLC 10 | GTCGAGCATGGCGAAGTTGG | |
| OMF 461 | GTACCACTGACCAGCTGACGCG | Confirm *espK* deletion, (2) |
| OMF 462 | ATAACTTACTTTCGAAAAACGCACTGGCAGCG | |
| ORB 124 | TTGGACGAGGCCGTCAAAG | *eccCb1* qRT-PCR, (18) |
| ORB 125 | CAGCGCCGACAATCATGTG | |
| esxA5′ | GGCAGCATCCAGCGCAATTC | *esxA* qRT-PCR, (20) |
| esxA3′ | GGTGGAGGACATTGCCTGAC | |
| ORS 148 | ACCCAGATCGACCAGGTTGAG | *esxB* qRT-PCR, (20) |
| ORS 149 | GAAGCCCATTTGCGAGGACAG | |
| ORB 134 | AGGCGACATTGGCTCTCAG | *MMAR_2894* qRT-PCR, this study. |
| ORB 135 | TTGAGCCGCCACATTGGAC | |
| ORB 117 | AAAGCCGATGGGTCCGTCAG | *ppe68* qRT-PCR, this study |
| ORB 118 | CCTCGTCATCCCAGTCGTCTTC | |
| ORB 119 | ACCAGCAGAAGGAGTGGATAC | *espB* qRT-PCR, this study |
| ORB 120 | GAGCTTGATGGCTTGCTCTTG | |
| OGC 245 | AAGCGGTGCAAACG | *espJ* qRT-PCR, this study |
| OGC 246 | TCGCCGATCTGTGTC | |
| OGC 247 | CATGTGGCTGGGTTG | *espK* qRT-PCR, this study |
| OGC 248 | AGCGGATCAGGGATG | |
| OLC 208 | GACGGCGTCTACAAGGTCTG | *espE* qRT-PCR, (6) |
| OLC 209 | CCGGAATGTTCGGGAGTAGG | |
| OLC 25 | TGACCCACGGCTCATTCAC | *espF* qRT-PCR, (6) |
| OLC 26 | GCGGCCGAGATCAGATTGTTG | |
| ORC 78 | GTGGACCTGACCTACATCCC | *espA* qRT-PCR, this study |
| ORC 79 | CAGGCTGACCAACTTCATCG | |
| ORS 126 | TGACGGAGAACCTGAAAGTG | *espC* qRT-PCR, this study |
| ORS 127 | TCGATACCGGCGTTATTGAG | |
| sigA-F | TCGAGGTGATCAACAAGCTG | *sigA* qRT-PCR, (3) |
| sigA-R | TGGATCTCCAGCACCTTCTC | |

**References**


1. W. Bitter *et al.*, Systematic genetic nomenclature for type VII secretion systems. *PLoS Pathog* **5**, e1000507 (2009).
2. R. M. Cronin, M. J. Ferrell, C. W. Cahir, M. M. Champion, P. A. Champion, Proteo-genetic analysis reveals clear hierarchy of ESX-1 secretion in *Mycobacterium marinum*. *Proc Natl Acad Sci U S A* **119**, e2123100119 (2022).
3. E. A. Williams *et al.*, A Nonsense Mutation in *Mycobacterium marinum* That Is Suppressible by a Novel Mechanism. *Infect Immun* **85** (2017).
4. T. Parish, N. G. Stoker, Use of a flexible cassette method to generate a double unmarked Mycobacterium tuberculosis tlyA plcABC mutant by gene replacement. *Microbiology* **146 ( Pt 8)**, 1969-1975 (2000).
5. O. A. Collars *et al.*, An N-acetyltransferase required for ESAT-6 N-terminal acetylation and virulence in *Mycobacterium marinum*. *mBio* **14**, e0098723 (2023).
6. A. E. Chirakos, K. R. Nicholson, A. Huffman, P. A. Champion, Conserved ESX-1 Substrates EspE and EspF Are Virulence Factors That Regulate Gene Expression. *Infect Immun* **88** (2020).
7. R. E. Bosserman, K. R. Nicholson, M. M. Champion, P. A. Champion, A New ESX-1 Substrate in *Mycobacterium marinum* That Is Required for Hemolysis but Not Host Cell Lysis. *J Bacteriol* **201** (2019).
8. K. G. Sanchez *et al.*, EspM Is a Conserved Transcription Factor That Regulates Gene Expression in Response to the ESX-1 System. *mBio* **11** (2020).
9. J. D. Sauer *et al.*, *Listeria monocytogenes* triggers AIM2-mediated pyroptosis upon infrequent bacteriolysis in the macrophage cytosol. *Cell host & microbe* **7**, 412-419 (2010).
10. F. Carlsson, S. A. Joshi, L. Rangell, E. J. Brown, Polar localization of virulence-related Esx-1 secretion in mycobacteria. *PLoS Pathog* **5**, e1000285 (2009).
11. S. M. Fortune *et al.*, Mutually dependent secretion of proteins required for mycobacterial virulence. *Proc Natl Acad Sci U S A* **102**, 10676-10681 (2005).
12. B. McLaughlin *et al.*, A mycobacterium ESX-1-secreted virulence factor with unique requirements for export. *PLoS Pathog* **3**, e105 (2007).
13. B. S. Jones *et al.*, The loss of the PDIM/PGL virulence lipids causes differential secretion of ESX-1 substrates in *Mycobacterium marinum*. *mSphere* **9**, e0000524 (2024).
14. J. W. Saelens *et al.*, An ancestral mycobacterial effector promotes dissemination of infection. *Cell* https://doi.org/10.1016/j.cell.2022.10.019 (2022).
15. M. E. Ritchie *et al.*, limma powers differential expression analyses for RNA-sequencing and microarray studies. *Nucleic acids research* **43**, e47 (2015).
16. B. S. Jones *et al.*, N-acetyltransferases required for iron uptake and aminoglycoside resistance promote virulence lipid production in *Mycobacterium marinum*. *Proc Natl Acad Sci U S A* **122**, e2502577122 (2025).
17. G. Viswanathan *et al.*, Granuloma Dual RNA-Seq Reveals Composite Transcriptional Programs Driven by Neutrophils and Necrosis within Tuberculous Granulomas. *bioRxiv* 10.1101/2025.04.26.650783 (2025).
18. R. E. Bosserman *et al.*, WhiB6 regulation of ESX-1 gene expression is controlled by a negative feedback loop in *Mycobacterium marinum*. *Proc Natl Acad Sci U S A* 10.1073/pnas.1710167114 (2017).
19. R. J. Prest, K. V. Korotkov, P. A. Champion, The regulatory functions of ESX-1 substrates, EspE and EspF, are separable from secretion. *J Bacteriol* **206**, e0027124 (2024).

20. R. E. Bosserman, C. R. Thompson, K. R. Nicholson, P. A. Champion, Esx Paralogs Are Functionally Equivalent to ESX-1 Proteins but Are Dispensable for Virulence in *Mycobacterium marinum*. *J Bacteriol* **200**, e00726-00717 (2018).
